# Supplementary material for: Life course exposures continually shape antibody profiles and risk of seroconversion to influenza
Source: PLoS Pathog. 2020 Jul 23;16(7):e1008635. doi: 10.1371/journal.ppat.1008635 (PMC7377380; doi:10.1371/journal.ppat.1008635)
Supplement: S1 Text — (DOCX) [file ppat.1008635.s001.docx]

## S1 Appendix.

### Effects of normalization of summary metrics

Results of AUC that included pre-birth strains (i.e. isolated before participants were born) showed similar age patterns to including only post-birth strains, except a lower ratio of average AUC among participants aged $\leq$20 years old to that among 40-70s (0.96 for baseline and 0.82 for follow-up) (S2 Fig). This is due to the relatively shorter exposure history of participants aged $\leq$20 years old compared to that among 40-70s (i.e. a smaller number of strains that could have been exposed to), and the lower titers to pre-birth strains (Figs. 2A-B and 3).

Non-normalized W_40_ illustrates an increasing trend as participants’ age grows until ~30 years old, which is older than the peaking age of nW_40_ (S2 Fig). Shorter exposure history of young children and lower responses to pre-birth strains contributed to the lower W_40_ compared to nW_40_. As a result, the drop in W_10_ among 40 - 70 years old is less compared to that in nW_40_.

Results of ATY that included pre-birth strains showed almost identical pattern to that of nATY (S2 Fig).

### Ceiling effects on changes in titers

We characterized the strain distribution among subgroups of titers, i.e. decrease, no change, two-fold increase and seroconversion and compared these distributions with the underlying strain distribution (S14 Fig). The underlying strain distribution was calculated by dividing the number of available titers for the strain by the total number of available titers for all strains in the examined dataset. In order to assess the ceiling effects, we performed this analysis to two separate subsets of titers that only include pre-existing titers $\leq1:80$ and $>1:80$ (S14 Fig), respectively. Results suggested that the strain distribution by changes in titers seemed not to be affected by the ceiling effects, with the most dynamic changes observed for recent strains.

### Effects of pre-existing immunity on seroconversion to recent strains

#### Univariable analysis and cross-correlations

We performed univariable logistic regressions of seroconversion to each of the four recent strains on the covariates included in the main analysis, i.e. age at baseline sampling, titer to strain *i*, titer to strain *i-1* and each summary metric of antibody profiles. Only titers to strain *i* and strain *i-1* were found to have negative associations with seroconversion to all four recent strains (S6 Table). In addition, we found negative associations between seroconversion to a given strain that were isolated after 1992 and titers to its antigenic relatives (S15A Fig) from univariable logistic regressions of seroconversion of strain *a* on titer to strain *b*. However, such associations were not significant after accounting for the titer to the examined outcome strain (S15B Fig). These results confirmed the protective effects of pre-existing, homologous titer on seroconversion.

We also found strong correlation between covariates that we included in the models to examine the effects of pre-existing immunity on seroconversion to four recent strains (S7 Table). We found titer to strain *i*, titer to strain *i-1* and each summary metric of antibody profiles was negatively associated with age at baseline sampling. The summary metrics of antibody profiles were positively associated with titer to strain *i*, which is likely driven by the cross-reactions between the tested strains.

#### nW_10_ and nATY

We also fitted Model 2 and 3 in Table 1 by changing the summary metrics of antibody profiles to nW_40_ and nATY. Except for nW10 for A/Victoria/2009, we found positive associations between nW_10_ or nATY and seroconversion to recent strains after adjusting for age at sampling, titer to strain *i* and strain *i-1* (S4 Table). However, including nW10 or nATY seems not always improve the model fit, especially when accounting for the non-linear effects of age (S6, S13 Figs).

#### Non-normalized AUC, nW_40,_ nW_10_ and ATY

We fitted Model 2 and 3 in Table 1 by changing the summary metrics of antibody profiles to non-normalized summary metric, i.e. AUC, nW_40_, nW_10_ and ATY. Specifically, we fitted logistic regressions to seroconversion on recent strains and adjusted age at sampling, titer to strain *i*, titer to strain *i-1* and one of the non-normalized summary metric of antibody profiles. Positive associations were only found for ATY for all four strains and W_40_ for A/Perth/2009, A/Victoria/2009 and A/Texas/2012 (S3-4 Tables). Non-normalized ATY and W40 were less likely to be affected by the pre-birth strains, as individuals are more likely to have weak but broad cross-reactions to strains that they could not have been exposed to.

#### Impact of age

We assumed a linear effect of age on seroconversion to recent strains in the main analysis (Table 1), and performed sensitivity analysis by assuming a non-linear effect of age. We fitted GAM with seroconversion to recent strains adjusted for age in a spline form, titer to strain *i*, titer to strain *i-1* and the summary metrics. We found consistent patterns in the association between age and seroconversion after adjusting for other covariates, however, these associations were not significant (S10 Fig) and did not affect our main outcome (S5 Table).

#### Performance of predictions in seroconversion to recent strains

We compared the predicted probability of seroconversion to four recent strains with the observed proportion of seroconversion among age groups binned by 10 years, in order to examine the model performance in predictions. We examined the uncertainty of prediction with the interquartile of predicted probability of each age group and derived 95% CI of observed proportion of seroconversion assuming a binomial distribution. Predictions and observations showed good consistency, and both were with great uncertainty (S16-17 Figs). There were two age groups with high predicted probability but low observed proportion of seroconversion when assuming a linear association between age and seroconversion (S16 Fig), which was due to the edge effect of age that can be accounted by a spline term on age (S17 Fig).

### Effects of vaccination against influenza

We included serum from all participants regardless of their vaccination status in the main analysis and used serum from participants who self-reported no previous vaccination against influenza in both visits in the sensitivity analysis (S8 Table), in particular summary metrics of antibody profiles and effects of pre-existing immunity on seroconversion to recent strains. Age patterns in summary metrics of antibody profiles of those unvaccinated participants (S11 Fig) were similar to results including all participants (Figs 2 and S3). Vaccination status of influenza seems not to affect the associations between pre-existing immunity and odds of seroconversion to recent strains (S9 Table).
