# Supplementary figures and images for: Life course exposures continually shape antibody profiles and risk of seroconversion to influenza
Source: PLoS Pathog. 2020 Jul 23;16(7):e1008635. doi: 10.1371/journal.ppat.1008635 (PMC7377380; doi:10.1371/journal.ppat.1008635)

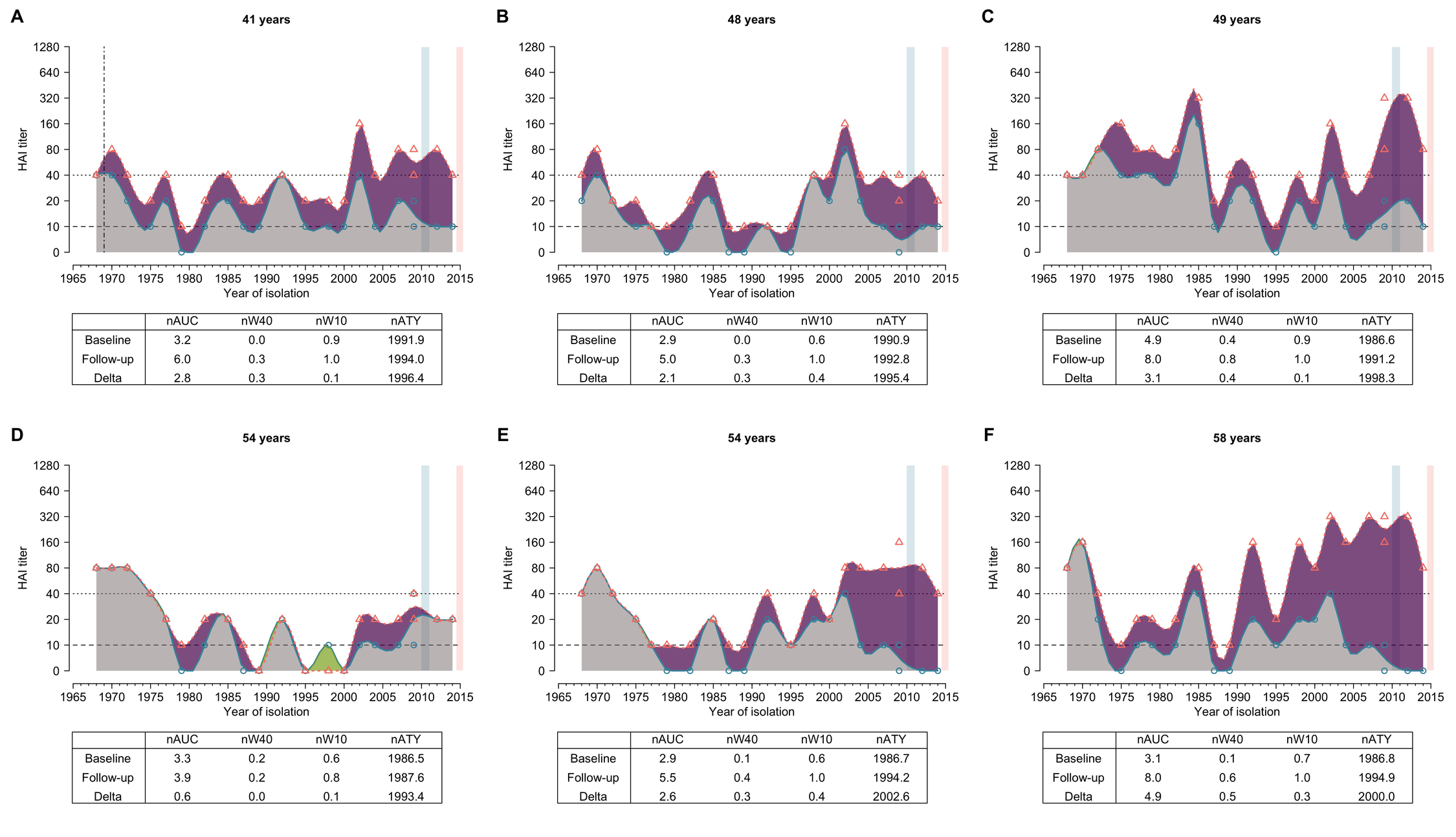

Supplement: S1 Fig — (A-F): Antibody profile for each representative individual aged 40–60 years. Blue circles and red triangles represent the HAI titers against the tested strains at baseline and follow-up visit, respectively. Blue and red solid lines represent the smoothed HAI titers for serum collected from baseline and follow-up visit, respectively. Smooth splines of HAI titers on circulating years are shown in this figure for illustration purposes and not used in the subsequent analysis. Grey areas represent the baseline antibody profile. Purple and green areas indicate the increase and decrease of HAI titer at follow-up visit compared to baseline, respectively. Blue and red vertical blocks represent the duration for baseline and follow-up visit, respectively. Vertical dotted-dashed lines indicate the year of birth of the individual. Dashed and dotted lines represent the titer of 1:10 (detectable cutoff) and 1:40 (protective cutoff), respectively. (TIF) [file ppat.1008635.s002.tif]

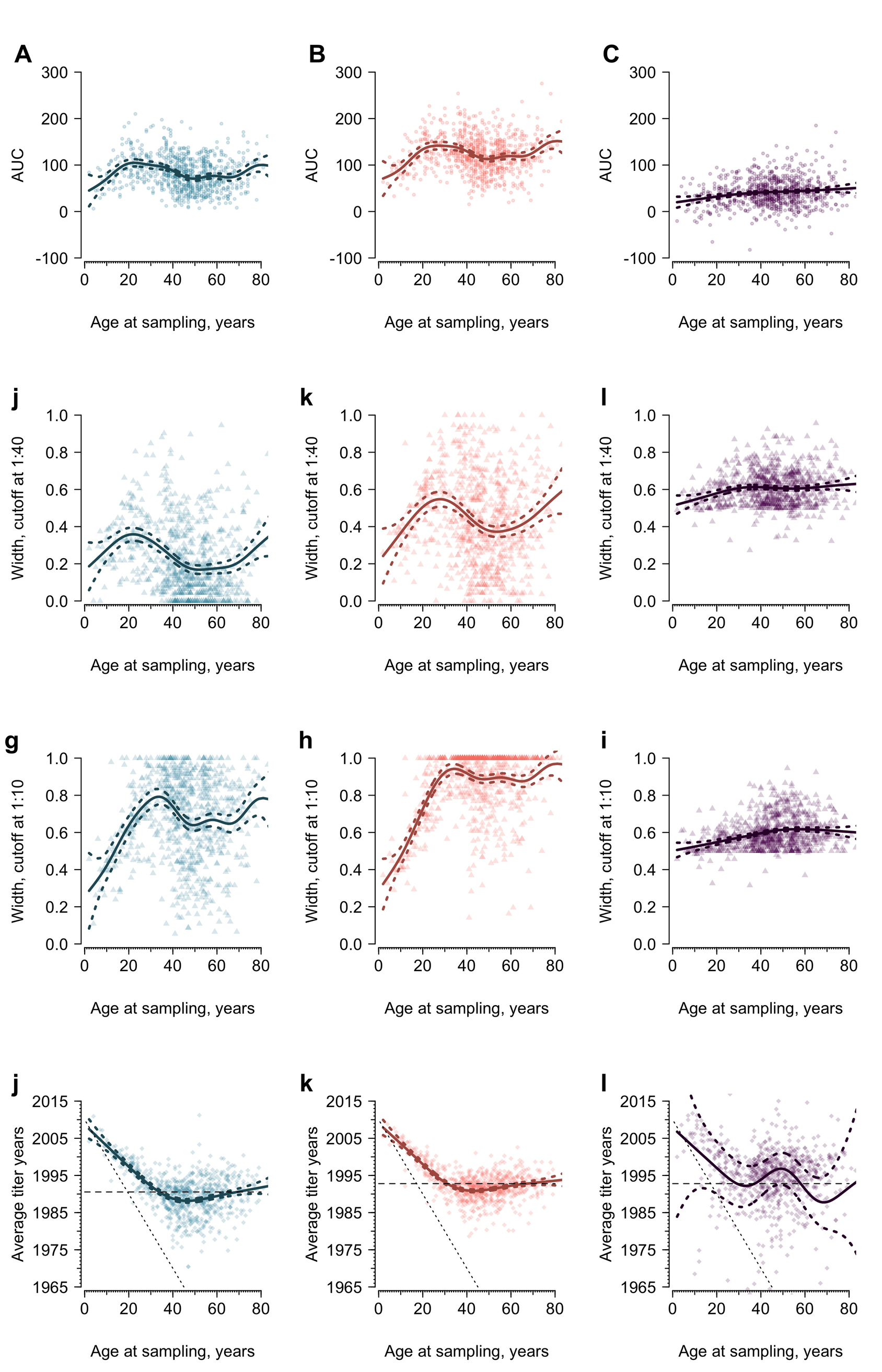

Supplement: S2 Fig — Blue and red represent the AUC for the baseline and follow-up visit, respectively. Purple indicates the differences of indicators between the two visits. Solid lines are predictions from gam and the colored dashed lines represent the corresponding 95% confidence intervals. The sloping black dotted lines in panel J to L indicate the year of birth of participants. The dashed lines in panel J to L indicate the unweighted average isolation year of all strains. (TIF) [file ppat.1008635.s003.tif]

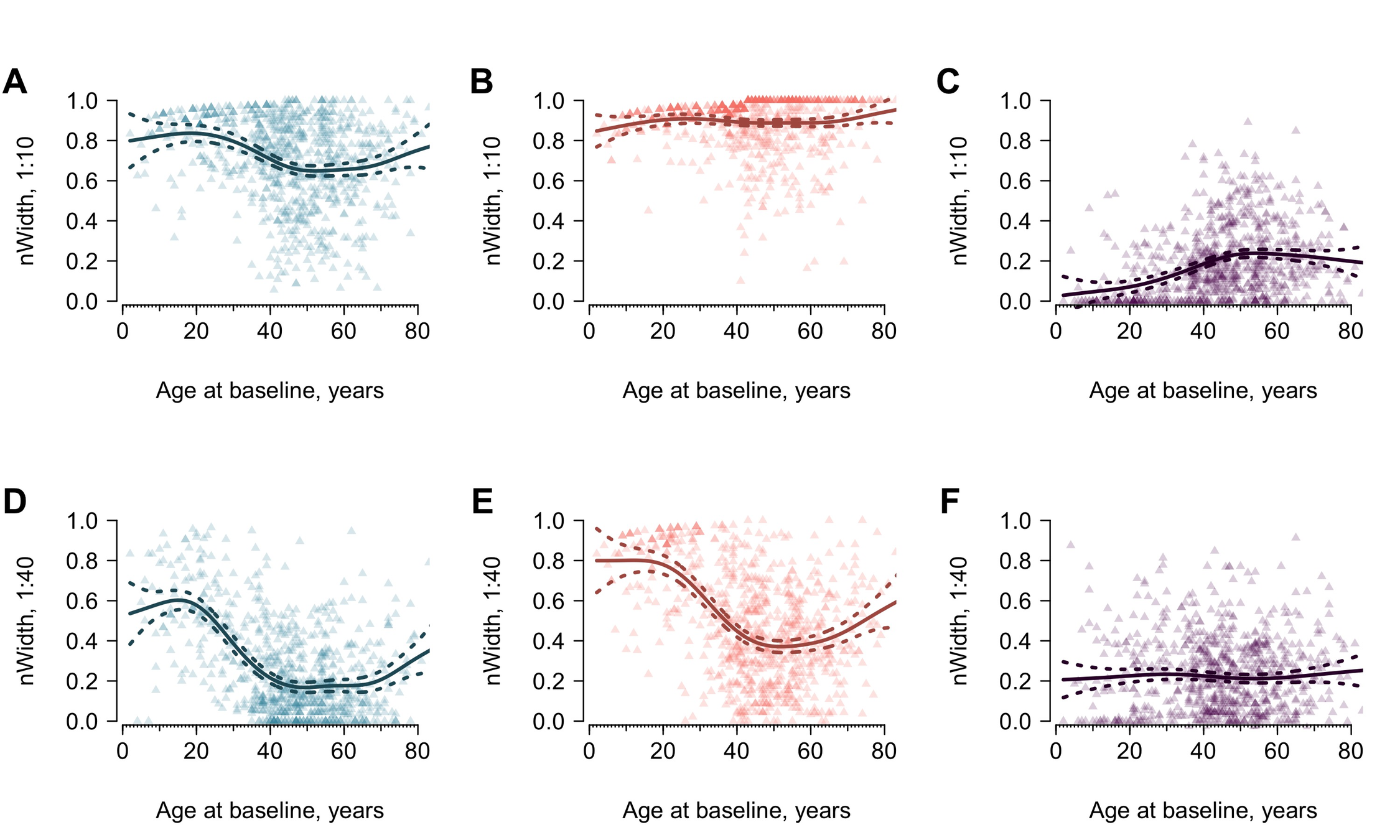

Supplement: S3 Fig — Widths were calculated using post-birth strains only. Panel A to C demonstrate width above titer 1:10, and Panel D to F demonstrate width above titer 1:40. Blue and red represent the indicators measured for serum collected in 2010 and 2014, respectively. Purple indicates the differences of indicators between the two visits. Solid lines are predictions from generalized additive model and the colored dashed lines represent the corresponding 95% confidence intervals. Results were calculated including all strains. (TIF) [file ppat.1008635.s004.tif]

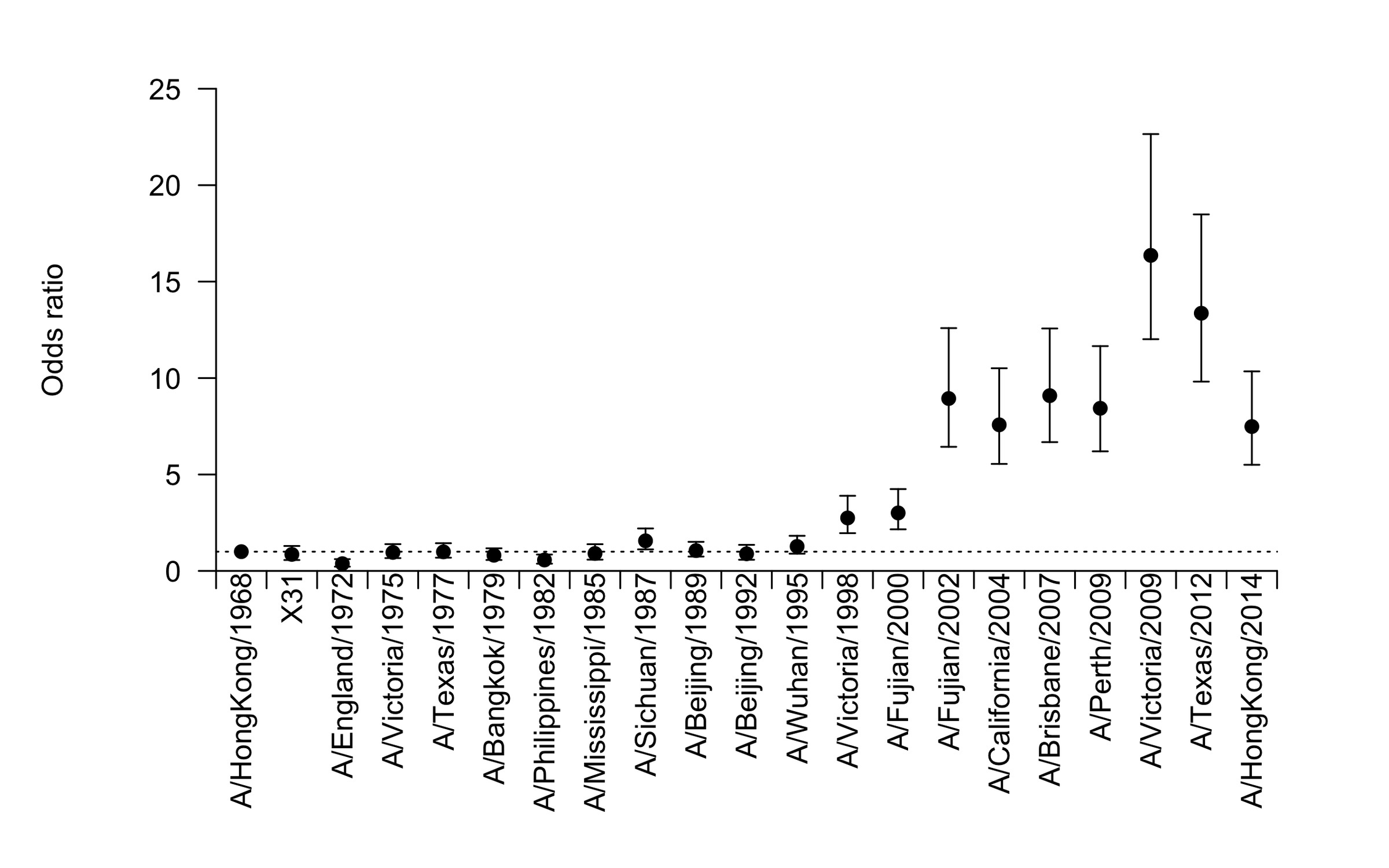

Supplement: S4 Fig — Logistic regression models were fitted using age at sampling, prior titer and strains to predict the seroconversion. Coefficients for H3N2 strains are shown in the figure. The A/HongKong/1968 strain was set as reference. (TIF) [file ppat.1008635.s005.tif]

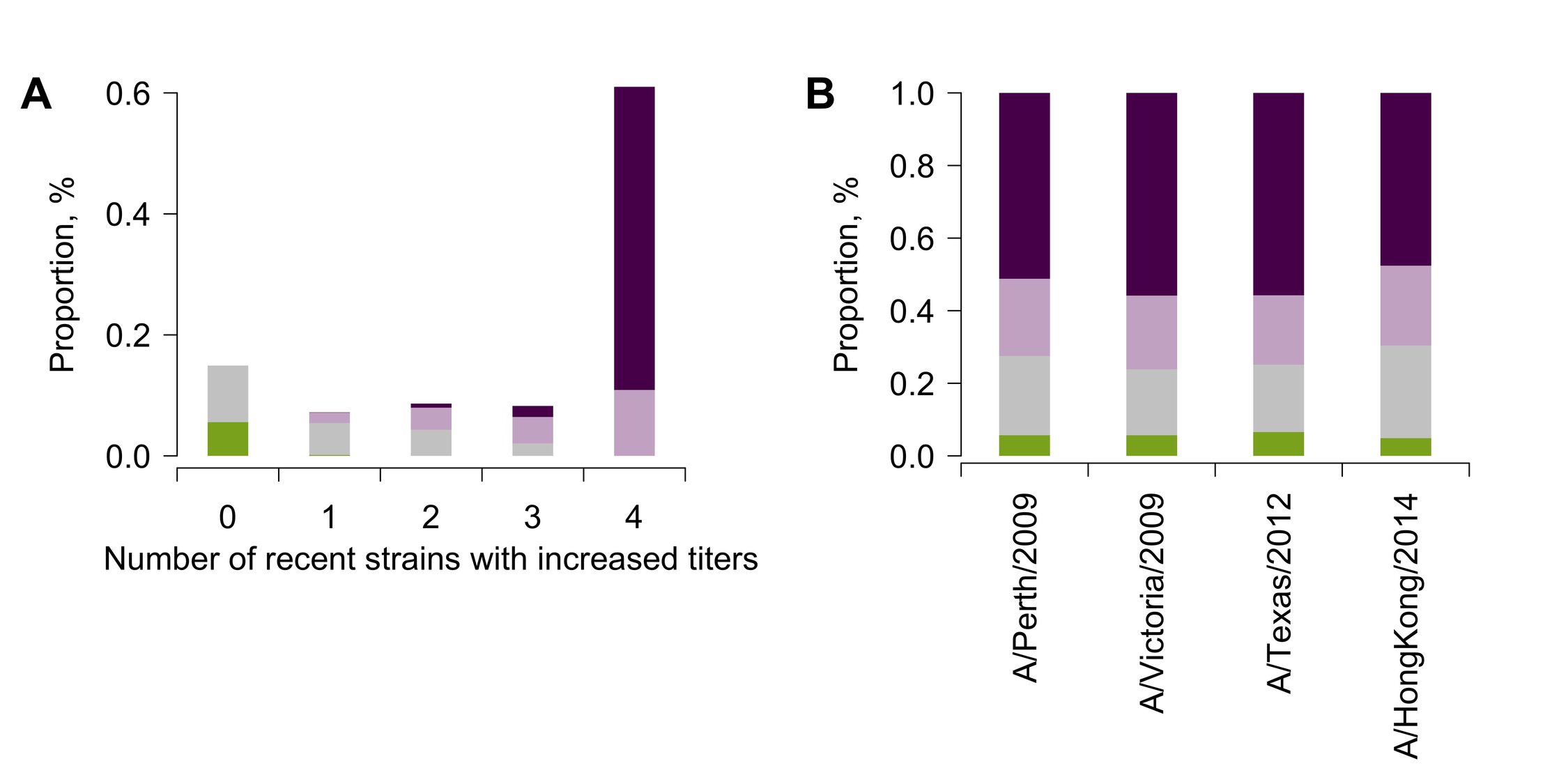

Supplement: S5 Fig — (A) Distribution of changes in titers against recent H3N2 strains by the number of strains with increased titers. (B) Distribution of changes in titers against recent H3N2 strains by individual strain. We divided the changes in titers into four categories, i.e. decrease (green), no change (grey), two-fold increase (light purple) and four-fold change (seroconversion, dark purple). (TIF) [file ppat.1008635.s006.tif]

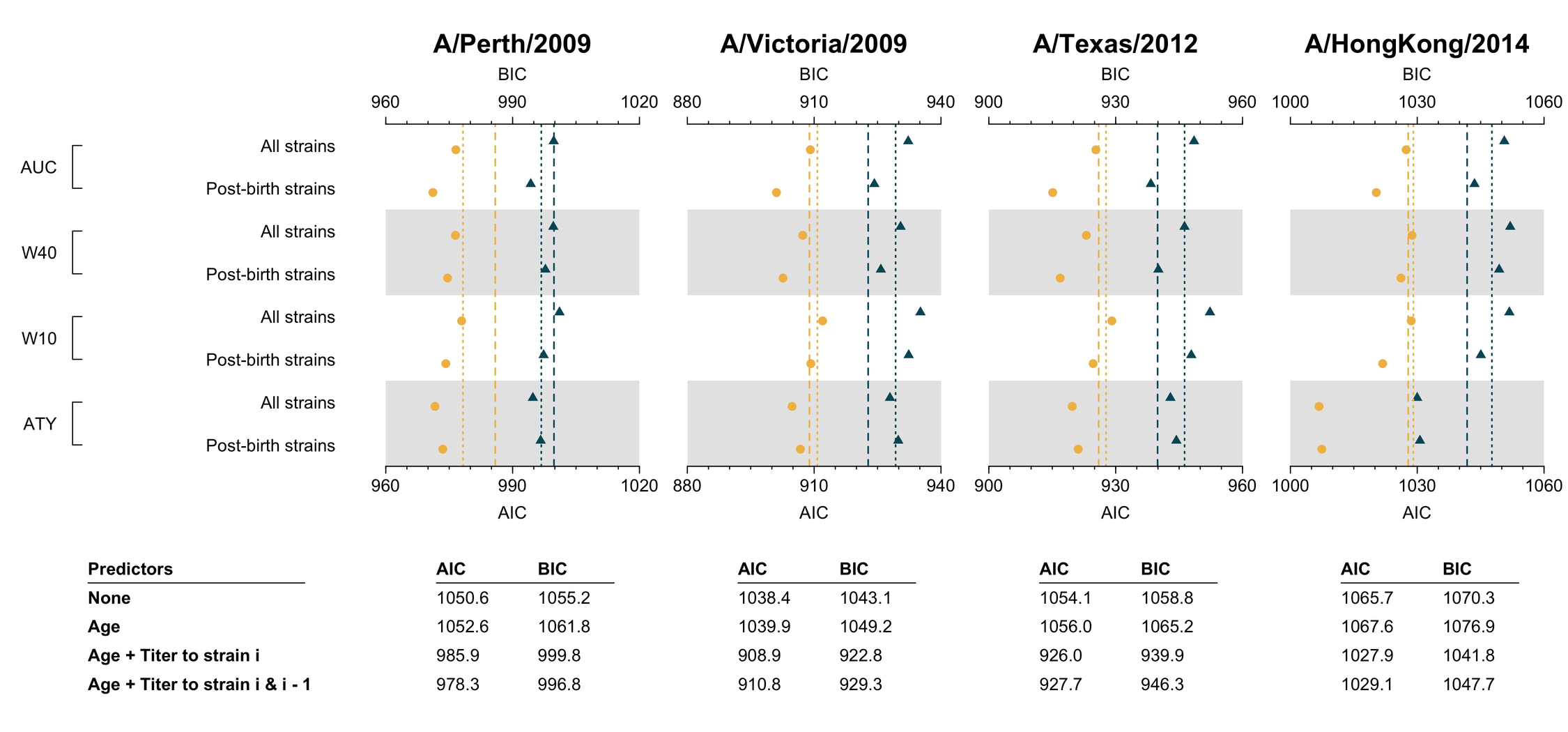

Supplement: S6 Fig — Yellow and blue represents AIC and BIC, respectively. Dashed lines represent the AIC/BIC for models that only included titer to the examined strain i. Dotted lines represent the AIC/BIC for models that included titers to the examined strain i and the prior strain i-1. Dots are AIC/BIC for models including additional predictor of pre-existing immunity of strains up to strain i-1. (TIF) [file ppat.1008635.s007.tif]

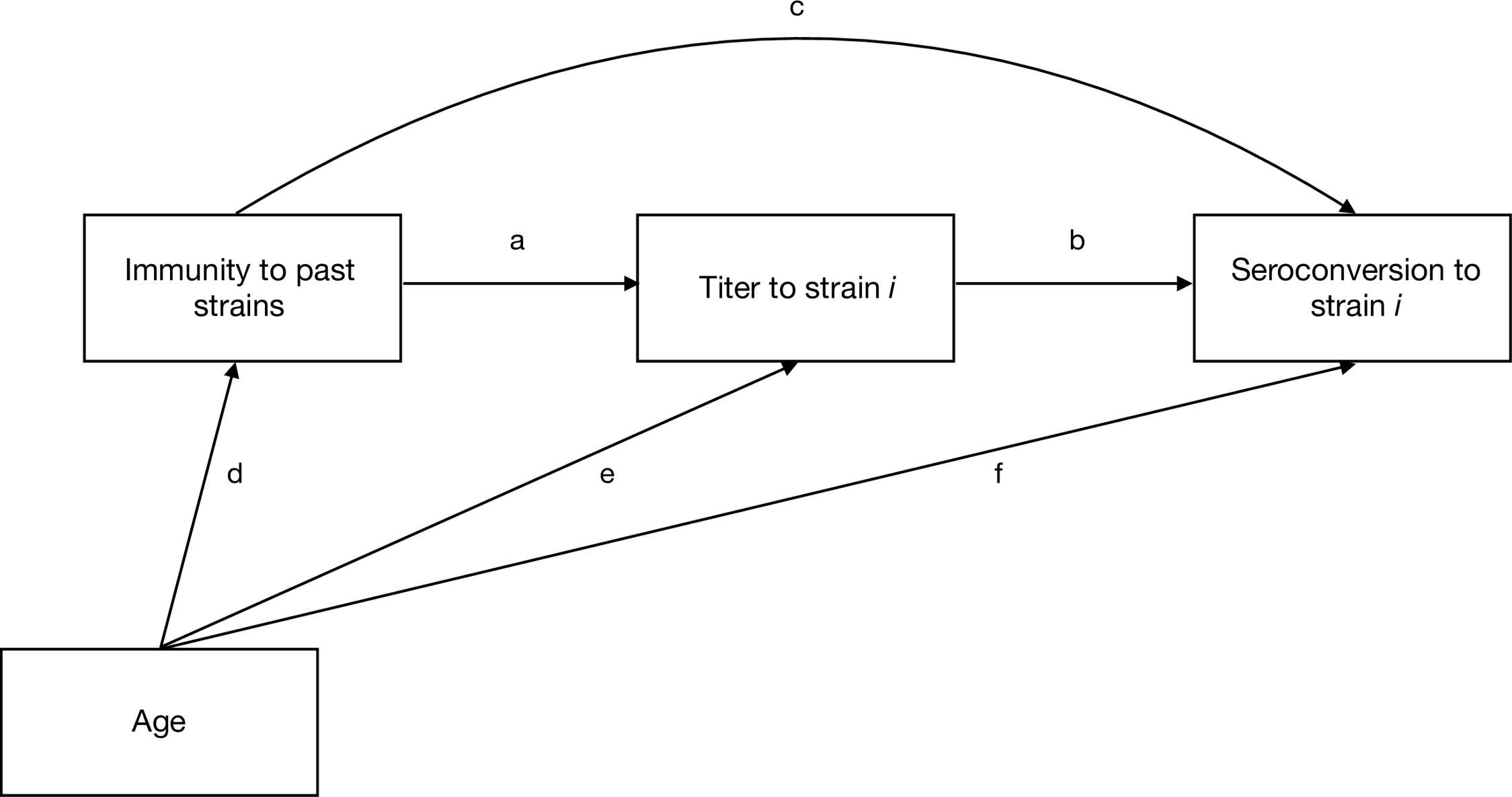

Supplement: S7 Fig — Indirect effect (path a path b): immune responses to previous strains have positive association between titer to strain i due to cross-reactions (path a), which has a negative association with seroconversion to strain i (path b). Direct effect (path c): effect of immune responses on seroconversion to strain i that was not mediated by titer to strain i. Total effect (path a path b + path c): combination of indirect effect and direct effect. Confounding effect (path d, e and f). (TIF) [file ppat.1008635.s008.tif]

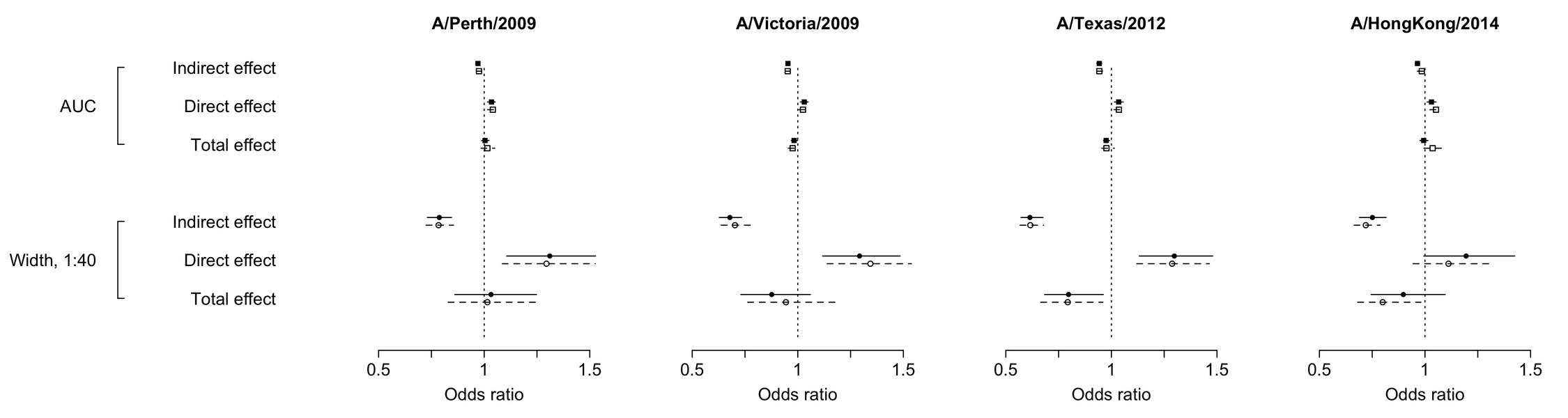

Supplement: S8 Fig — Solid lines and filled squares represent the estimates from mediation analysis that did not consider interactions. Dashed lines and open circles represent the estimates from mediation analysis that considered interactions. (TIF) [file ppat.1008635.s009.tif]

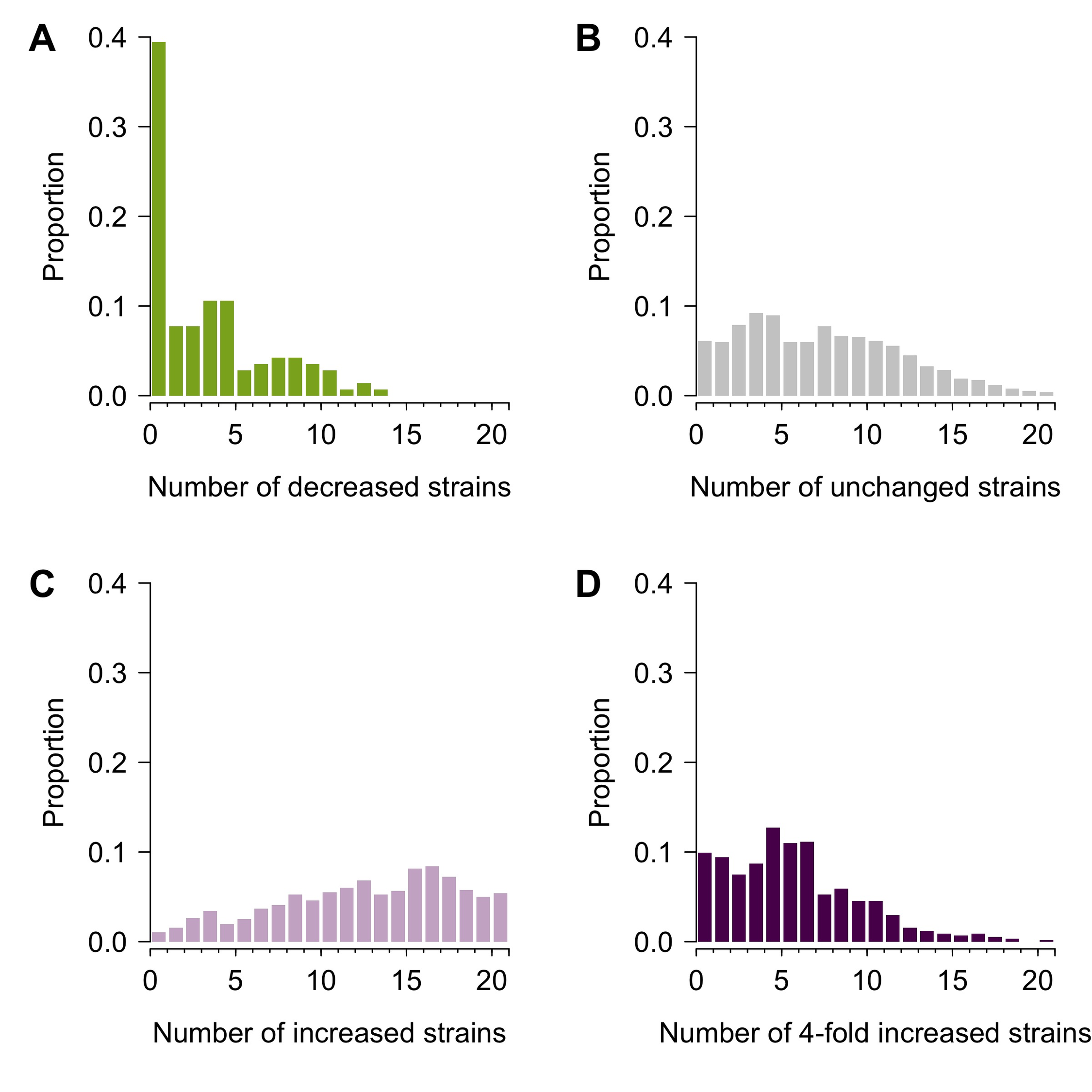

Supplement: S9 Fig — Results are shown by subgroups of participants who had decreased (A), unchanged (B), increased (C) and four-fold increased (D) titers between the two visits, respectively. (TIF) [file ppat.1008635.s010.tif]

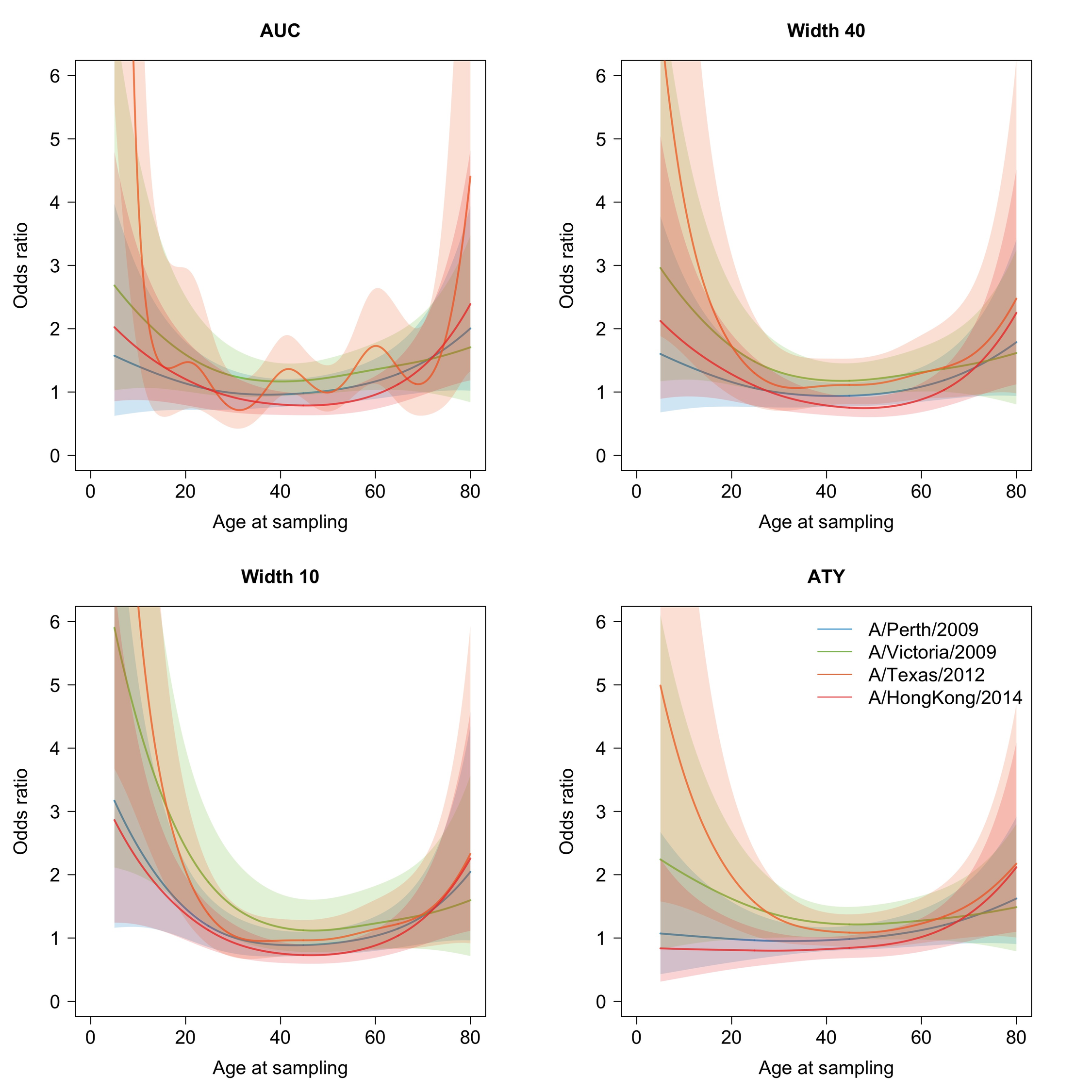

Supplement: S10 Fig — Models has been adjusted for titer to strain i, titer to strain i-1, and summary metrics. (TIF) [file ppat.1008635.s011.tif]

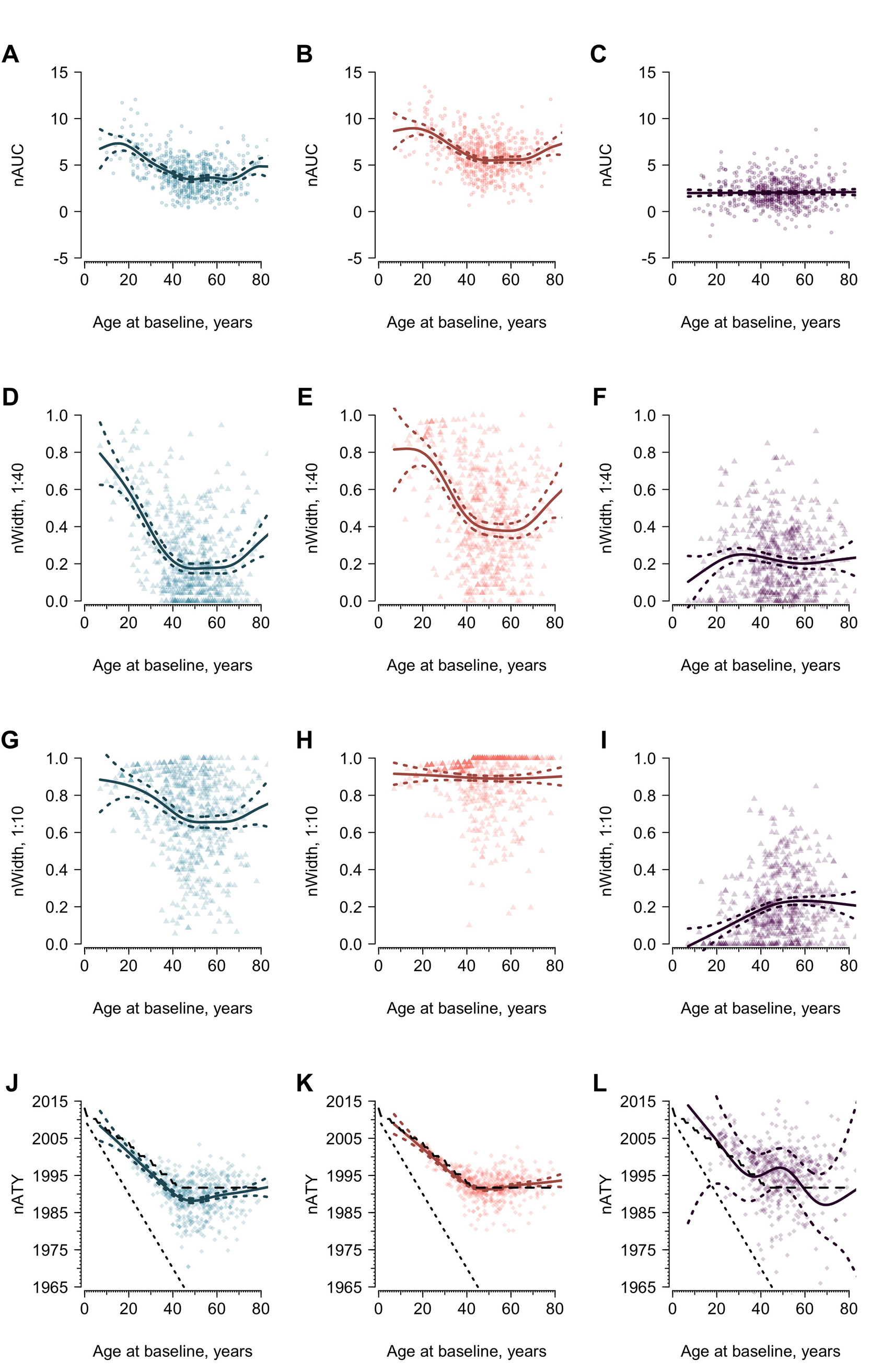

Supplement: S11 Fig — Blue and red represent the AUC for the baseline and follow-up visit, respectively. Purple indicates the differences of indicators between the two visits. Solid lines are predictions from gam and the colored dashed lines represent the corresponding 95% confidence intervals. The sloping black dotted lines in panel J to L indicate the year of birth of participants. The dashed lines in panel J to L indicate the unweighted average isolation year of post-birth strains. (TIF) [file ppat.1008635.s012.tif]

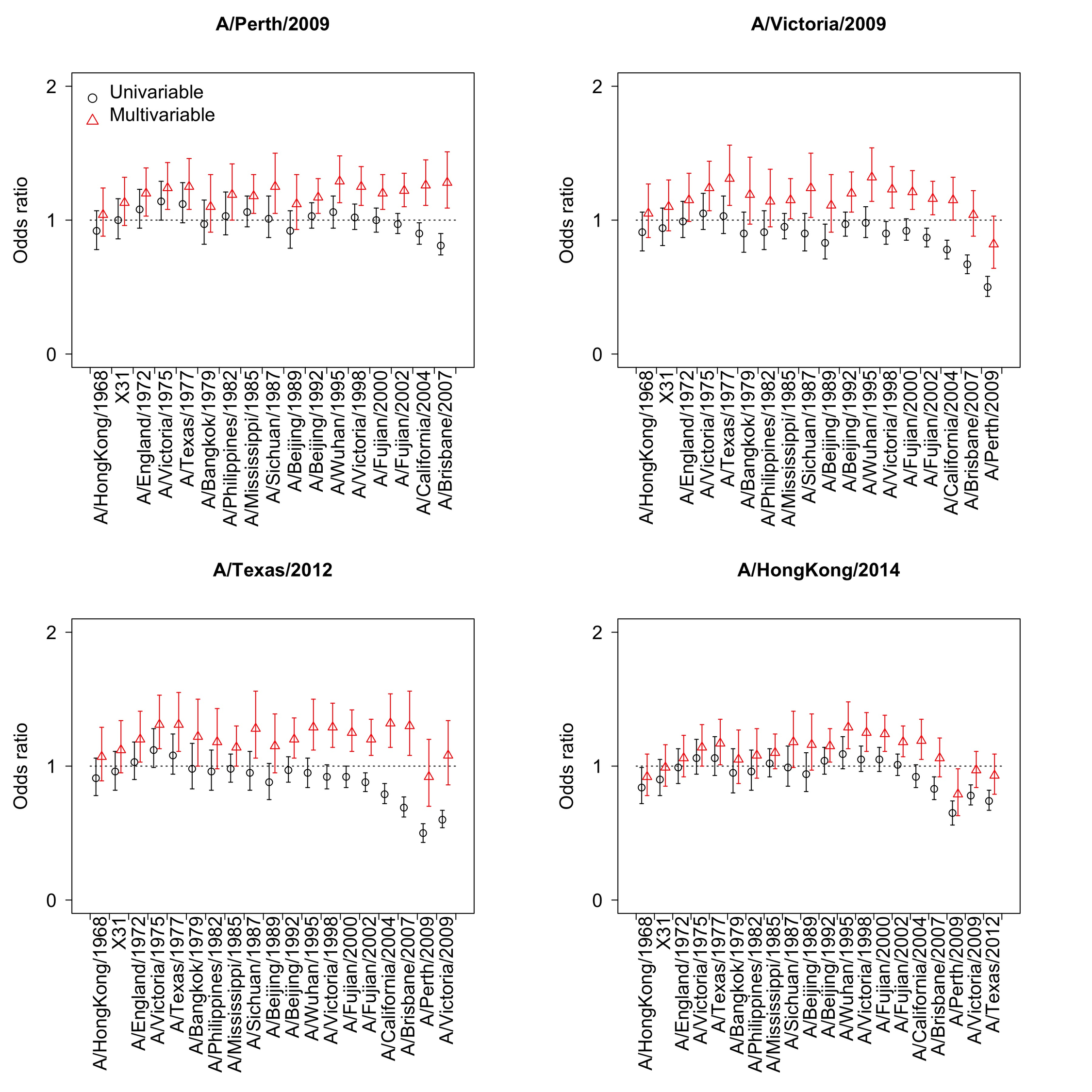

Supplement: S12 Fig — Univariable coefficient (black) is estimated from univariable logistic regression of seroconversion to strain i on pre-existing titer to the strain listed in x-axis. Multivariable coefficient (red) is estimated from multivariable logistic regression of seroconversion to a strain i on pre-existing titer to the strain listed in x-axis, adjusting for age at sampling and titer to strain i and i-1. (TIF) [file ppat.1008635.s013.tif]

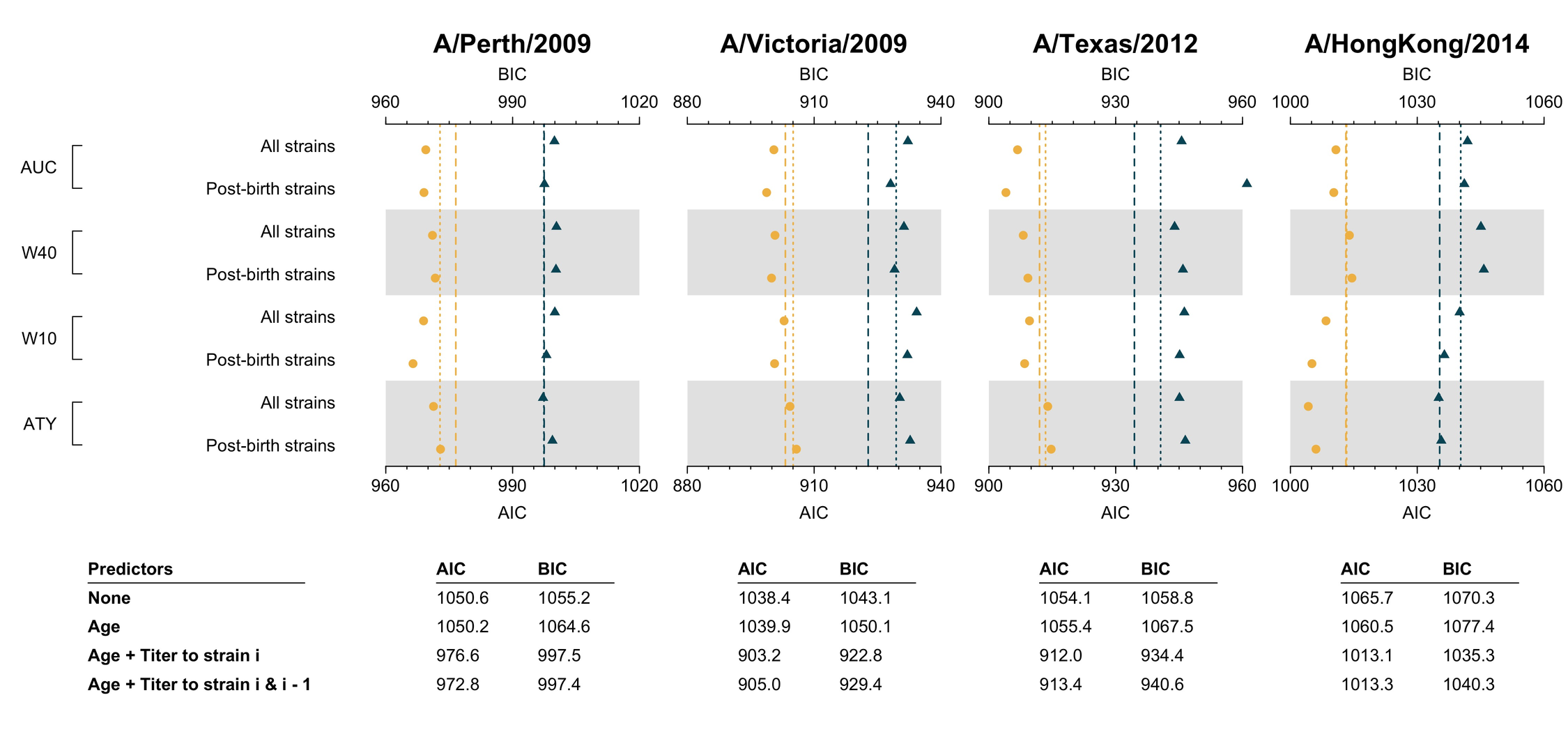

Supplement: S13 Fig — Yellow and blue represents AIC and BIC, respectively. Dashed lines represent the AIC/BIC for models that only included titer to the examined strain i. Dotted lines represent the AIC/BIC for models that included titers to the examined strain i and the prior strain i-1. Dots are AIC/BIC for models including additional predictor of pre-existing immunity of strains up to strain i-1. (TIF) [file ppat.1008635.s014.tif]

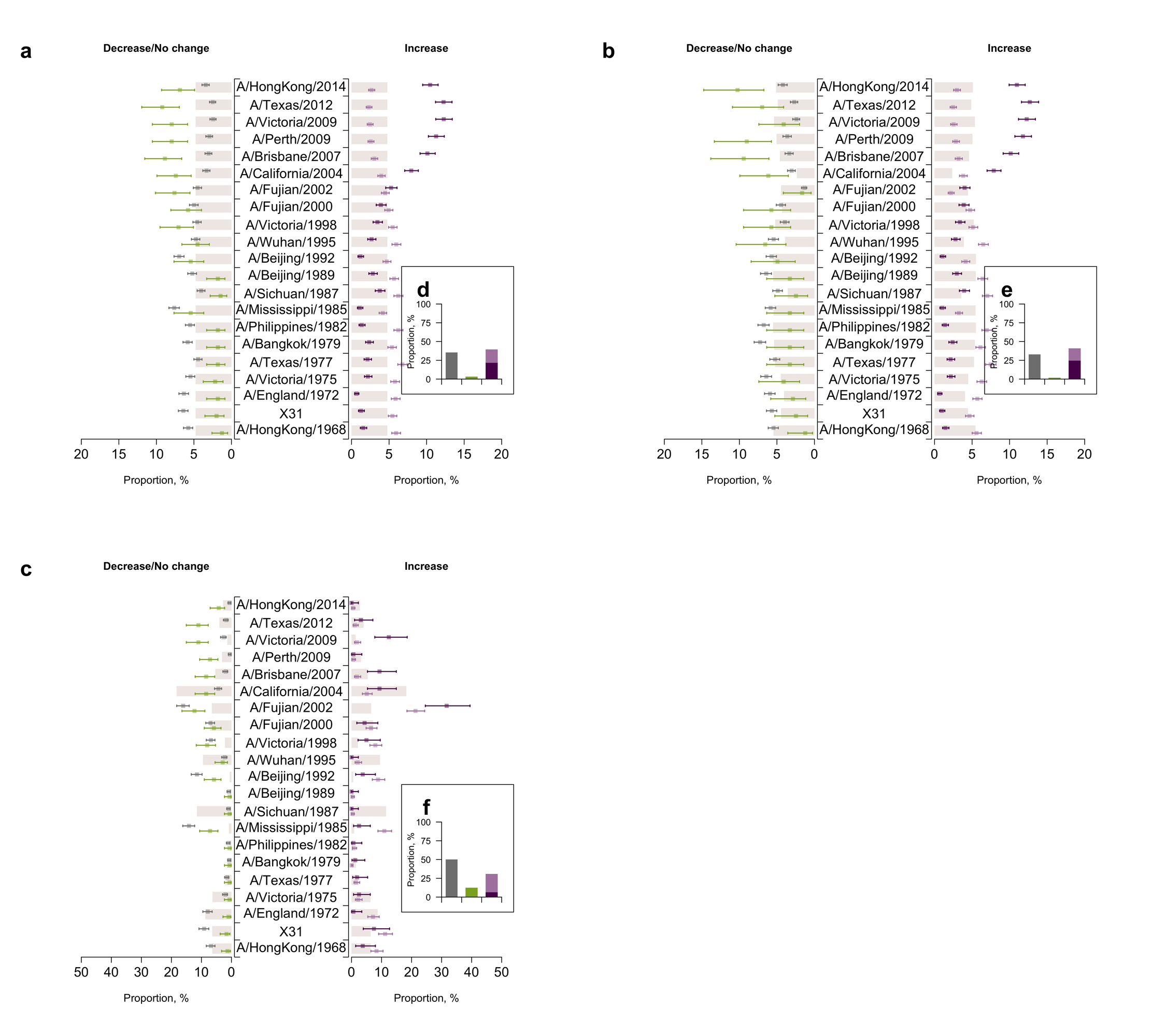

Supplement: S14 Fig — We divided the examined data (i.e. all data, or pre-existing titer is greater or less than 1:80) on titer changes into four subgroups, i.e. decreased (green), unchanged (grey), any fold increase (light purple, including four-fold or more increase) and four-fold or more increase (dark purple). Colored points and lines represent the distribution of H3N2 strains within each subgroup. Colored bars represent the distribution of H3N2 strains regardless of titer changes for the examined data. (A) all data; (B) a subset contains pre-existing titers ≤ 1:40; (C) a subset contains pre-existing titers > 1:40. Insets D to F illustrate the distribution of changes in titers between two visits. (TIF) [file ppat.1008635.s015.tif]

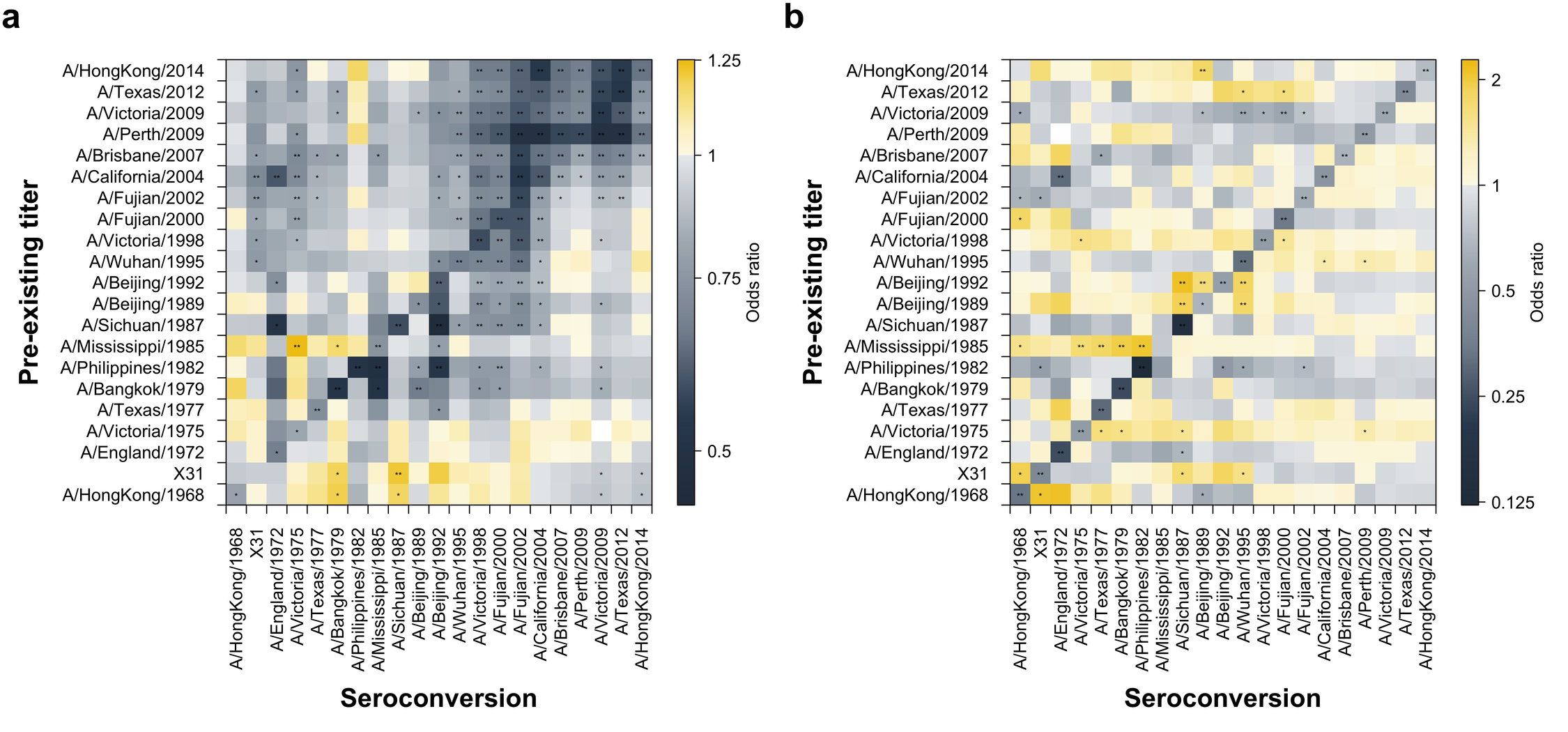

Supplement: S15 Fig — Univariable analysis of pre-existing titer on seroconversion. Coefficient was derived from univariable logistic regression of seroconversion to strain in x-axis on pre-existing titer to a strain listed in y-axis. Each cell represents an individual model. (B) Multivariable analysis of pre-existing titers on seroconversion. Coefficients were derived from multivariable logistic regression of seroconversion to a strain in x-axis on age at sampling and pre-existing titers to all strains listed in y-axis. Each column represents an individual model. Each cell within a column represents the association between pre-existing titer to the strain listed in the y-axis on the seroconversion to strain in x-axis, after adjusting for the pre-existing titers to the rest of the twenty strains. Asterisks indicate p ≤ 0.01. (TIF) [file ppat.1008635.s016.tif]

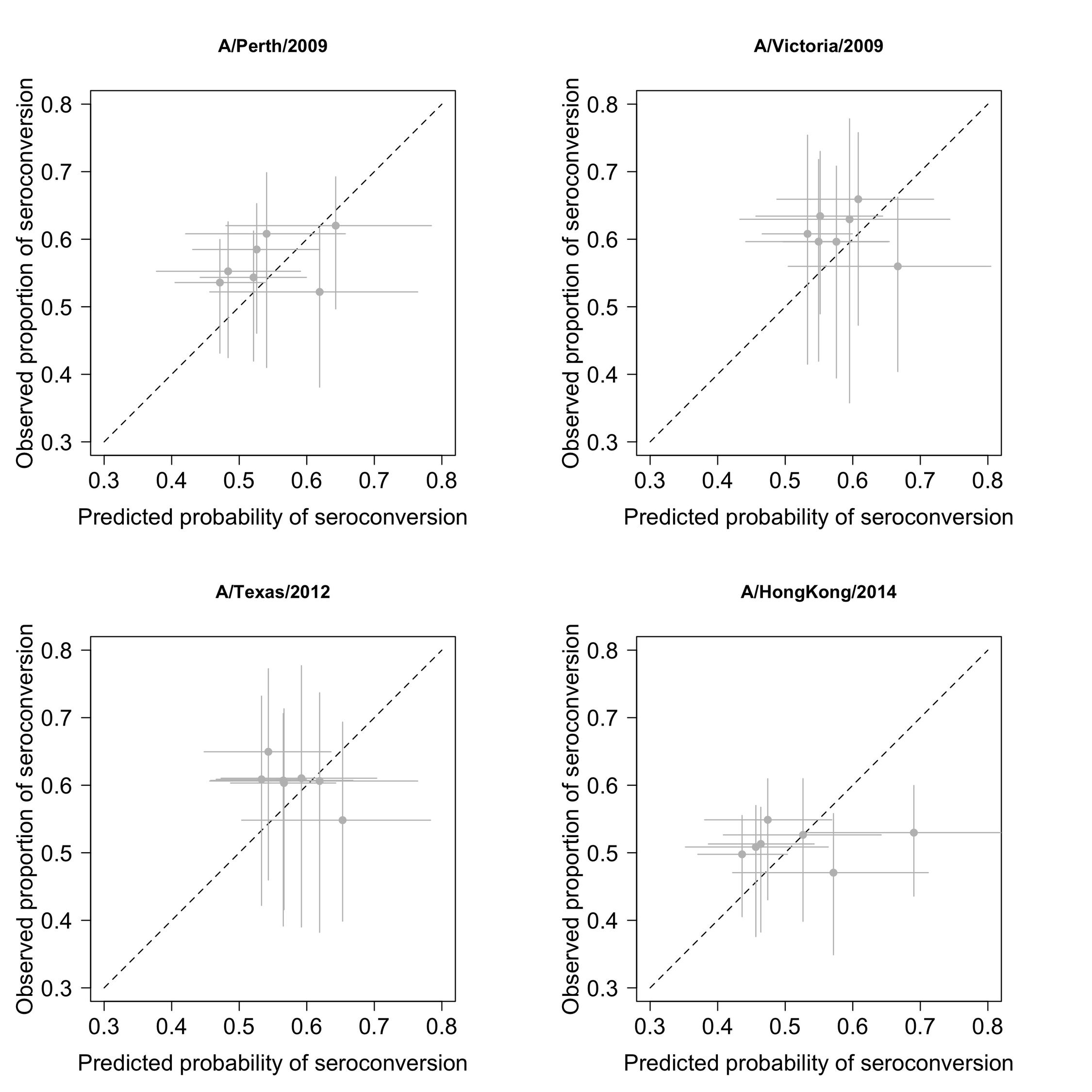

Supplement: S16 Fig — Models are fitted with a linear term on age, i.e. models used in Table 1. Age group was binned by 10 years. Horizontal lines represent the interquartile of predicted probability of seroconversion for the age group. Vertical lines represent 95% CI of the observed proportion of seroconversion derived from binomial distribution. (TIF) [file ppat.1008635.s017.tif]

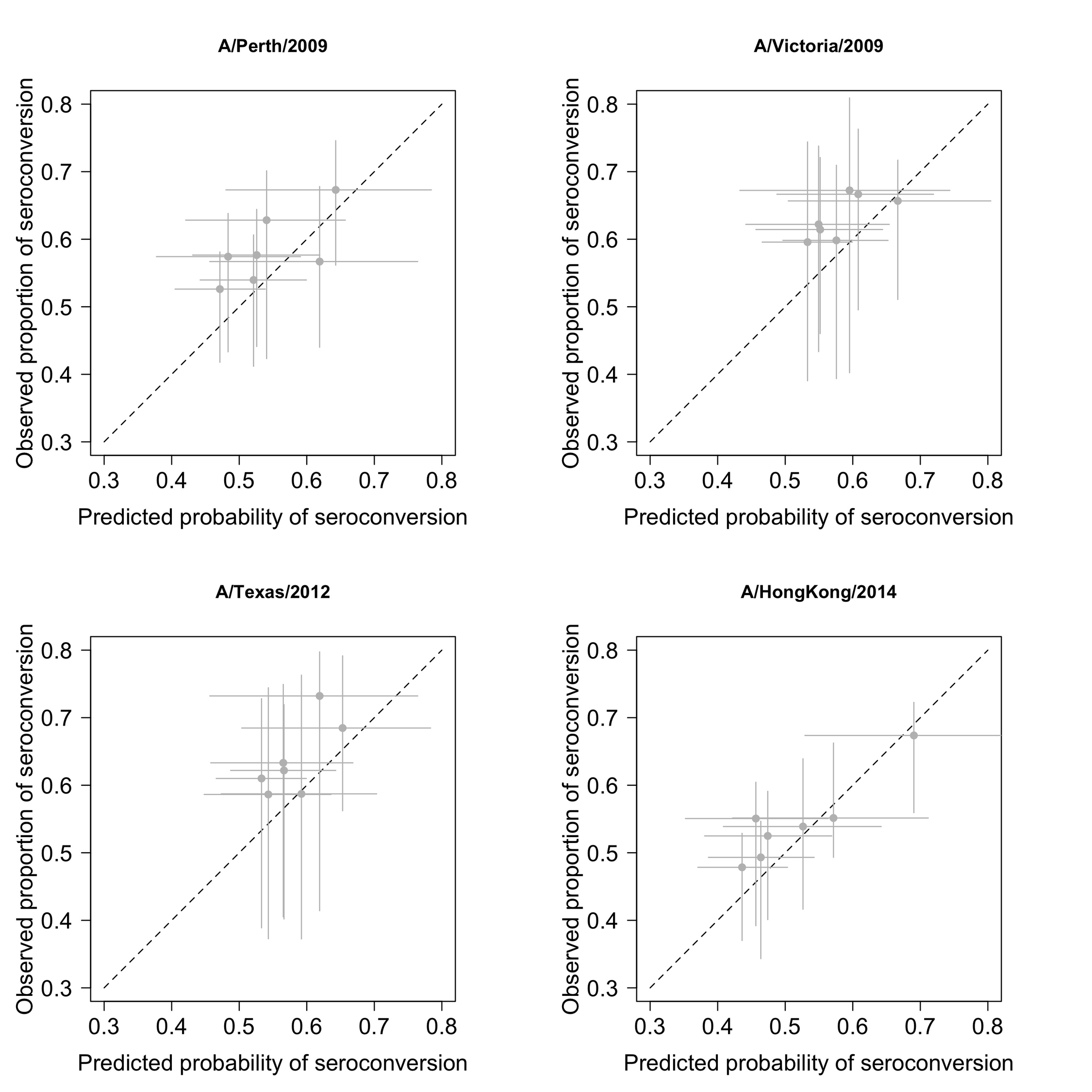

Supplement: S17 Fig — Models are fitted with a spline term on age, i.e. models used in S9 Table. Age group was binned by 10 years. Horizontal lines represent the interquartile of predicted probability of seroconversion for the age group. Vertical lines represent 95% CI of the observed proportion of seroconversion derived from binomial distribution. (TIF) [file ppat.1008635.s018.tif]
