# Supplementary material for: Life course exposures continually shape antibody profiles and risk of seroconversion to influenza
Source: PLoS Pathog. 2020 Jul 23;16(7):e1008635. doi: 10.1371/journal.ppat.1008635 (PMC7377380; doi:10.1371/journal.ppat.1008635)
Supplement: S1 Table — (DOCX) [file ppat.1008635.s019.docx]

S1 Table. Comparison of demographic characteristics of participants.

|  | **Serum Available**  **(N, %)** | **Baseline** | | | **Follow-up** | | |
| --- | --- | --- | --- | --- | --- | --- | --- |
|  |  | **Unavailable**  **(N, %)** | **P^a^** | **Total**  **(N, %)** | **Unavailable**  **(N, %)** | **P^a^** | **Total**  **(N, %)** |
| **Total** | 777 | 1044 |  | 1821 | 1264 |  | 2039 |
| **Sex** |  |  |  |  |  |  |  |
| Male | 408 (52.5) | 531 (50.9) | 0.52 | 939 (51.6) | 630 (49.8) | 0.26 | 1042 (51.1) |
| Female | 369 (47.5) | 513 (49.1) |  | 882 (48.4) | 634 (50.2) |  | 997 (48.9) |
| **Age group, years** |  |  |  |  |  |  |  |
| < 10 | 11 (1.4) | 49 (4.7) | <0.01 | 60 (3.3) | 77 (6.1) | <0.01 | 79 (3.9) |
| 10-19 | 44 (5.7) | 143 (13.7) |  | 187 (10.3) | 119 (9.4) |  | 151 (7.4) |
| 20-29 | 83 (10.7) | 130 (12.5) |  | 213 (11.7) | 191 (15.1) |  | 260 (12.8) |
| 30-39 | 102 (13.1) | 130 (12.5) |  | 232 (12.7) | 160 (12.7) |  | 230 (11.3) |
| 40-49 | 233 (30.0) | 182 (17.4) |  | 415 (22.8) | 199 (15.7) |  | 401 (19.7) |
| 50-59 | 166 (21.4) | 142 (13.6) |  | 308 (16.9) | 207 (16.4) |  | 406 (19.9) |
| $\geq$ 60 | 138 (17.8) | 268 (25.7) |  | 406 (22.3) | 311 (24.6) |  | 512 (25.1) |
| **Employment status** |  |  |  |  |  |  |  |
| Full Time | 237 (30.5) | 265 (25.4) | <0.01 | 502 (27.6) | 421 (33.3) | <0.01 | 696 (34.1) |
| Self Employed | 97 (12.5) | 103 (9.9) |  | 200 (11.0) | 163 (12.9) |  | 323 (15.8) |
| Retired | 76 (9.8) | 142 (13.6) |  | 218 (12.0) | 249 (19.7) |  | 382 (18.7) |
| Student | 52 (6.7) | 166 (15.9) |  | 218 (12.0) | 185 (14.6) |  | 221 (10.8) |
| Homemaker | 90 (11.6) | 109 (10.4) |  | 199 (10.9) | 117 (9.3) |  | 207 (10.2) |
| Unemployed | 118 (15.2) | 160 (15.3) |  | 278 (15.3) | 90 (7.1) |  | 140 (6.9) |
| Other | 107 (13.8) | 99 (9.5) |  | 206 (11.3) | 39 (3.1) |  | 69 (3.4) |
| **Years since last influenza vaccination** | | |  |  |  |  |  |
| $<1$ | 10 (1.3) | 30 (2.9) | 0.04 | 40 (2.2) | 8 (0.6) | <0.01 | 12 (0.6) |
| 1 | 24 (3.1) | 39 (3.7) |  | 63 (3.5) | 11 (0.9) |  | 13 (0.6) |
| 2 – 5 | 17 (2.2) | 34 (3.3) |  | 51 (2.8) | 17 (1.3) |  | 19 (0.9) |
| $>5$ | 36 (4.6) | 59 (5.7) |  | 95 (5.2) | 34 (2.7) |  | 47 (2.3) |
| Never | 597 (76.8) | 738 (70.7) |  | 1335 (73.3) | 1131 (89.5) |  | 1865 (91.2) |
| Unknown/unsure | 93 (12.0) | 144 (13.8) |  | 237 (13.0) | 63 (5.0) |  | 89 (4.4) |

^a^Chi-squared test.
