# Supplementary material for: Life course exposures continually shape antibody profiles and risk of seroconversion to influenza
Source: PLoS Pathog. 2020 Jul 23;16(7):e1008635. doi: 10.1371/journal.ppat.1008635 (PMC7377380; doi:10.1371/journal.ppat.1008635)
Supplement: S2 Table — (DOCX) [file ppat.1008635.s020.docx]

S2 Table. Geometric mean titer of tested H3N2 strains.

| **Strain** | **All tested strains** | | **Post-birth strains** | | | **Pre-birth strains** | | |  |
| --- | --- | --- | --- | --- | --- | --- | --- | --- | --- |
|  | Baseline | Follow-up | | Baseline | Follow-up | | Baseline | Follow-up | |
| A/HongKong/1968 | 23.7 (22.0, 25.4) | 36.4 (33.7, 39.2) | | 36.8 (34.2, 39.5) | 59.1 (55.2, 63.3) | | 11.3 (10.2, 12.5) | 16.1 (14.3, 18.1) | |
| X31 | 27.2 (25.2, 29.4) | 39.9 (36.7, 43.4) | | 44.1 (41.2, 47.3) | 68.0 (63.3, 73.0) | | 9.7 (8.7, 10.8) | 12.9 (11.4, 14.6) | |
| A/England/1972 | 22.3 (20.7, 23.9) | 32.1 (29.8, 34.7) | | 30.0 (27.9, 32.2) | 45.3 (42.2, 48.5) | | 10.0 (8.8, 11.4) | 12.8 (11.0, 14.8) | |
| A/Victoria/1975 | 12.1 (11.4, 12.9) | 19.4 (18.0, 20.7) | | 14.3 (13.3, 15.4) | 24.1 (22.4, 26.0) | | 6.5 (6.0, 7.0) | 8.6 (7.7, 9.6) | |
| A/Texas/1977 | 13.1 (12.3, 13.9) | 22.0 (20.6, 23.5) | | 14.1 (13.2, 15.0) | 24.9 (23.3, 26.6) | | 9.5 (8.3, 10.9) | 13.2 (11.2, 15.6) | |
| A/Bangkok/1979 | 7.7 (7.4, 8.1) | 12.3 (11.6, 12.9) | | 8.0 (7.6, 8.4) | 13.3 (12.6, 14.1) | | 6.5 (6.0, 7.1) | 8.5 (7.6, 9.5) | |
| A/Philippines/1982 | 11.4 (10.8, 12.0) | 17.7 (16.8, 18.8) | | 12.2 (11.5, 12.8) | 19.6 (18.5, 20.8) | | 7.7 (6.8, 8.6) | 9.9 (8.5, 11.5) | |
| A/Mississippi/1985 | 38.5 (35.5, 41.6) | 51.2 (47.1, 55.6) | | 46.3 (42.9, 49.9) | 62.2 (57.6, 67.2) | | 10.5 (8.5, 13.0) | 13.0 (10.3, 16.5) | |
| A/Sichuan/1987 | 9.2 (8.7, 9.6) | 16.7 (15.7, 17.7) | | 9.0 (8.5, 9.4) | 16.8 (15.8, 17.8) | | 11.0 (8.8, 13.6) | 16.1 (12.3, 21.0) | |
| A/Beijing/1989 | 8.5 (8.1, 8.9) | 14.0 (13.2, 14.8) | | 8.6 (8.2, 9.0) | 14.5 (13.7, 15.4) | | 7.2 (6.0, 8.5) | 8.8 (7.2, 10.7) | |
| A/Beijing/1992 | 32.9 (30.5, 35.6) | 45.5 (42.2, 49.2) | | 34.8 (32.2, 37.6) | 48.8 (45.3, 52.6) | | 12.9 (8.9, 18.8) | 14.4 (9.7, 21.2) | |
| A/Wuhan/1995 | 13.4 (12.5, 14.2) | 21.9 (20.5, 23.4) | | 13.4 (12.5, 14.2) | 22.3 (20.9, 23.8) | | 14.9 (9.7, 22.9) | 14.9 (10.1, 22.0) | |
| A/Victoria/1998 | 27.7 (25.7, 29.9) | 46.8 (43.6, 50.2) | | 27.6 (25.6, 29.7) | 46.7 (43.5, 50.1) | | 34.8 (18.1, 67.0) | 52.8 (28.2, 98.7) | |
| A/Fujian/2000 | 25.9 (23.9, 28.0) | 43.9 (40.7, 47.3) | | 26.1 (24.1, 28.2) | 44.0 (40.8, 47.4) | | 16.8 (7.2, 39.1) | 37.8 (17.6, 80.9) | |
| A/Fujian/2002 | 71.8  (65.7, 78.3) | 133.1  (123.0, 144.1) | | 72.1  (66.0, 78.7) | 133.7  (123.5, 144.7) | | 65.6  (11.2, 383.0) | 118.9  (26.0, 543.3) | |
| A/California/2004 | 20.6 (19.0, 22.2) | 47.1 (43.6, 50.9) | | 20.6 (19.0, 22.2) | 47.1 (43.6, 50.8) | | 25.2 (0.7, 907.9) | 63.5  (1.8, 2287.8) | |
| A/Brisbane/2007 | 14.3 (13.3, 15.4) | 40.0 (36.8, 43.4) | | 14.3 (13.3, 15.4) | 39.9 (36.7, 43.3) | | Not available | Not available | |
| A/Perth/2009 | 9.1 (8.6, 9.7) | 30.9 (28.4, 33.5) | | 9.1 (8.6, 9.7) | 30.9 (28.4, 33.5) | | Not available | Not available | |
| A/Victoria/2009 | 17.5 (16.3, 18.9) | 67.6 (62.3, 73.3) | | 17.5 (16.3, 18.9) | 67.6 (62.3, 73.3) | | Not available | Not available | |
| A/Texas/2012 | 13.3 (12.4, 14.3) | 50.6 (46.5, 55.0) | | 13.3 (12.4, 14.3) | 50.6 (46.5, 55.0) | | Not available | Not available | |
| A/HongKong/2014 | 9.6 (9.0, 10.2) | 28.1 (25.9, 30.4) | | 9.6 (9.0, 10.2) | 28.1 (25.9, 30.4) | | Not available | Not available | |
| Overall | 17.1 (16.8, 17.4) | 33.0 (32.4, 33.6) | | 18.3 (18.0, 18.7) | 36.9 (36.2, 37.5) | | 9.6 (9.2, 10.0) | 12.7 (12.1, 13.4) | |
