# Supplementary material for: Life course exposures continually shape antibody profiles and risk of seroconversion to influenza
Source: PLoS Pathog. 2020 Jul 23;16(7):e1008635. doi: 10.1371/journal.ppat.1008635 (PMC7377380; doi:10.1371/journal.ppat.1008635)
Supplement: S3 Table — (DOCX) [file ppat.1008635.s021.docx]

S3 Table. Associations between pre-existing immunity and seroconversion to four recent strains, using titers to all tested strains.

|  | **A/Perth/2009** | **A/Victoria/2009** | **A/Texas/2012** | **A/HongKong/2014** |
| --- | --- | --- | --- | --- |
| **Model 1** |  |  |  |  |
| Age at sampling | 1.00 (0.99, 1.01) | 0.99 (0.98, 1.00) | 0.99 (0.98, 1.00)* | 1.00 (0.99, 1.01) |
| Titer to strain $i$^a^ | 0.43 (0.34, 0.54)* | 0.51 (0.42, 0.60)* | 0.49 (0.38, 0.61)* | 0.70 (0.56, 0.85)* |
| Titer to strain $i$-*1^a^* | 1.30 (1.10, 1.55)* | 1.03 (0.87, 1.22) | 1.06 (0.85, 1.32) | 0.93 (0.79, 1.09) |
| Deviance explained | 7.5% | 12.9% | 12.6% | 4.0% |
| **Model 2** |  |  |  |  |
| Age at sampling | 1.00 (0.99, 1.01) | 0.99 (0.98, 1.00) | 0.99 (0.98, 1.00) | 1.00 (0.99, 1.01) |
| Titer to strain $i$ | 0.43 (0.34, 0.54)* | 0.49 (0.41, 0.58)* | 0.47 (0.37, 0.60)* | 0.68 (0.55, 0.84)* |
| Titer to strain $i$-*1* | 1.26 (1.06, 1.50)* | 1.02 (0.86, 1.21) | 1.00 (0.80, 1.26) | 0.89 (0.75, 1.05) |
| AUC^b^ | 1.01 (1.00, 1.01) | 1.01 (1.00, 1.01) | 1.01 (1.00, 1.01)* | 1.01 (1.00, 1.01) |
| Deviance explained | 7.8% | 13.3% | 13.0% | 4.4% |
| **Model 3** |  |  |  |  |
| Age at sampling | 1.00 (0.99, 1.01) | 0.99 (0.98, 1.00) | 0.99 (0.98, 1.00) | 1.00 (0.99, 1.01) |
| Titer to strain $i$ | 0.43 (0.34, 0.54)* | 0.49 (0.41, 0.58)* | 0.47 (0.37, 0.60)* | 0.68 (0.55, 0.84)* |
| Titer to strain $i$-*1* | 1.26 (1.06, 1.50)* | 1.01 (0.85, 1.19) | 1.01 (0.80, 1.26) | 0.90 (0.76, 1.07) |
| Width, cut off 1:40^b^ | 2.86 (0.99, 8.40) | 3.85 (1.24, 12.31)* | 4.15 (1.40, 12.63)* | 2.15 (0.80, 5.80) |
| Deviance explained | 7.8% | 13.4% | 13.2% | 4.2% |

^a^ Strain *i* refers to the strain that was examined for seroconversion, and strain *i-1* refers to the most recent strain isolated prior to strain *i*. E.g. when using seroconversion to A/Perth/2009 as outcome, strain *i* and *i-1* will be A/Perth/2009 and A/Brisbane/2007, respectively.

^b^ Metrics were calculated using titers to strains isolated after 1968 and before the year that strain *i* was isolated, regardless of whether the strain circulated before or after the birth of the participant.
