# Supplementary material for: Life course exposures continually shape antibody profiles and risk of seroconversion to influenza
Source: PLoS Pathog. 2020 Jul 23;16(7):e1008635. doi: 10.1371/journal.ppat.1008635 (PMC7377380; doi:10.1371/journal.ppat.1008635)
Supplement: S4 Table — (DOCX) [file ppat.1008635.s022.docx]

S4 Table. Associations between pre-existing average titer year or width above detectable threshold and seroconversion to four recent strains.

|  | **A/Perth/2009** | **A/Victoria/2009** | **A/Texas/2012** | **A/HongKong/2014** |
| --- | --- | --- | --- | --- |
| **All tested strains** | | | | |
| **Model A** |  |  |  |  |
| Age at sampling | 1.01 (0.99, 1.02) | 1.00 (0.99, 1.01) | 1.00 (0.99, 1.01) | 1.01 (1.00, 1.02) |
| Titer to strain $i$^a^ | 0.43 (0.34, 0.54)* | 0.51 (0.42, 0.60)* | 0.44 (0.34, 0.56)* | 0.67 (0.54, 0.82)* |
| Titer to strain $i$-*1*^a^ | 1.21 (1.01, 1.44)* | 0.96 (0.81, 1.15) | 1.05 (0.85, 1.32) | 0.82 (0.69, 0.98)* |
| ATY^b^ | 1.06 (1.02, 1.10)* | 1.06 (1.02, 1.11)* | 1.06 (1.02, 1.10)* | 1.09 (1.05, 1.13)* |
| Deviance explained | 8.3% | 13.7% | 13.5% | 6.3% |
| **Model B** |  |  |  |  |
| Age at sampling | 1.00 (0.99, 1.01) | 0.99 (0.98, 1.00)* | 0.99 (0.98, 1.00)* | 0.99 (0.98, 1.00) |
| Titer to strain $i$^a^ | 0.43 (0.34, 0.54)* | 0.50 (0.42, 0.60)* | 0.48 (0.38, 0.61)* | 0.69 (0.56, 0.85)* |
| Titer to strain $i$-*1*^a^ | 1.28 (1.08, 1.53)* | 1.03 (0.87, 1.22) | 1.04 (0.83, 1.30) | 0.90 (0.76, 1.07) |
| Width, cut off 1:10^b^ | 1.92 (0.83, 4.49) | 1.50 (0.60, 3.72) | 1.41 (0.60, 3.27) | 1.86 (0.87, 4.00) |
| Deviance explained | 7.7% | 13.0% | 12.6% | 4.2% |
| **Post-birth strains** | | | | |
| **Model A** |  |  |  |  |
| Age at sampling | 1.01 (1.00, 1.02) | 1.00 (0.99, 1.01) | 1.00 (0.99, 1.01) | 1.01 (1.00, 1.02)* |
| Titer to strain $i$^a^ | 0.44 (0.35, 0.55)* | 0.51 (0.42, 0.60)* | 0.44 (0.34, 0.56)* | 0.67 (0.54, 0.83)* |
| Titer to strain $i$-*1*^a^ | 1.21 (1.02, 1.45)* | 0.97 (0.81, 1.16) | 1.05 (0.85, 1.32) | 0.82 (0.69, 0.98)* |
| ATY^c^ | 1.05 (1.01, 1.09)* | 1.05 (1.01, 1.10)* | 1.06 (1.02, 1.10)* | 1.09 (1.05, 1.13)* |
| Deviance explained | 8.1% | 13.0% | 13.4% | 6.2% |
| **Model B** |  |  |  |  |
| Age at sampling | 1.00 (0.99, 1.01) | 0.99 (0.98, 1.00) | 0.99 (0.98, 1.00) | 1.00 (0.99, 1.01) |
| Titer to strain $i$^a^ | 0.43 (0.34, 0.53)* | 0.49 (0.41, 0.58)* | 0.47 (0.37, 0.60)* | 0.68 (0.55, 0.84)* |
| Titer to strain $i$-*1*^a^ | 1.27 (1.07, 1.51)* | 1.03 (0.87, 1.22) | 1.00 (0.80, 1.26) | 0.87 (0.73, 1.03) |
| Width, cut off 1:10^c^ | 2.78 (1.23, 6.32)* | 2.33 (0.97, 5.66) | 2.78 (1.14, 6.80)* | 3.72 (1.59, 8.81)* |
| Deviance explained | 8.0% | 13.2% | 13.1% | 4.9% |

^a^ Strain *i* refers to the strain that was examined for seroconversion, and strain *i-1* refers to the most recent strain isolated prior to strain *i*. E.g. when using seroconversion to A/Perth/2009 as outcome, strain *i* and *i-1* will be A/Perth/2009 and A/Brisbane/2007, respectively.

^b^ Metrics were calculated using titers to strains isolated after 1968 and before the year that strain *i* was isolated, regardless of whether the strain circulated before or after the birth of the participant.

^c^ Metrics were calculated using titers to strains isolated after the birth of the participants and before the year that strain *i-1* was isolated. Adjustment was then performed by standardizing the metrics with the number of post-birth strains.
