# Supplementary material for: Life course exposures continually shape antibody profiles and risk of seroconversion to influenza
Source: PLoS Pathog. 2020 Jul 23;16(7):e1008635. doi: 10.1371/journal.ppat.1008635 (PMC7377380; doi:10.1371/journal.ppat.1008635)
Supplement: S5 Table — (DOCX) [file ppat.1008635.s023.docx]

S5 Table. Associations between and pre-existing immunity and seroconversion to four recent strains, considering the non-linear impact of age.

|  | **A/Perth/2009** | **A/Victoria/2009** | **A/Texas/2012** | **A/HongKong/2014** |
| --- | --- | --- | --- | --- |
| **Model 1** |  |  |  |  |
| Titer to strain $i$^a^ | 0.44 (0.35, 0.55)* | 0.52 (0.43, 0.62)* | 0.45 (0.35, 0.58)* | 0.69 (0.56, 0.85)* |
| Titer to strain $i$-*1*^a^ | 1.25 (1.05, 1.48)* | 0.98 (0.83, 1.16) | 1.09 (0.87, 1.36) | 0.90 (0.76, 1.06) |
| Deviance explained | 8.2% | 13.7% | 14.3% | 5.8% |
| **Model 2** |  |  |  |  |
| Titer to strain $i$ | 0.43 (0.34, 0.54)* | 0.49 (0.40, 0.58)* | 0.41 (0.32, 0.53)* | 0.67 (0.54, 0.83)* |
| Titer to strain $i$-*1* | 1.18 (0.99, 1.41) | 0.94 (0.79, 1.12) | 0.99 (0.79, 1.26) | 0.84 (0.70, 1.00) |
| AUC^b^ | 1.12 (1.03, 1.23)* | 1.16 (1.05, 1.27)* | 1.21 (1.08, 1.36)* | 1.13 (1.02, 1.25)* |
| Deviance explained | 8.8% | 14.5% | 16.4% | 6.3% |
| **Model 3** |  |  |  |  |
| Titer to strain $i$ | 0.43 (0.35, 0.55)* | 0.50 (0.41, 0.59)* | 0.43 (0.34, 0.55)* | 0.68 (0.55, 0.84)* |
| Titer to strain $i$-*1* | 1.21 (1.01, 1.44)* | 0.94 (0.79, 1.13) | 1.03 (0.82, 1.30) | 0.88 (0.74, 1.04) |
| Width, cut off 1:40^b^ | 2.38 (0.96, 5.89) | 3.84 (1.44, 10.24)* | 3.72 (1.32, 10.51)* | 1.68 (0.64, 4.43) |
| Deviance explained | 8.5% | 14.4% | 15.1% | 5.9% |

^a^ Strain *i* refers to the strain that was examined for seroconversion, and strain *i-1* refers to the most recent strain isolated prior to strain *i*. E.g. when using seroconversion to A/Perth/2009 as outcome, strain *i* and *i-1* will be A/Perth/2009 and A/Brisbane/2007, respectively.

^b^ Metrics were calculated using titers to strains isolated after the birth of the participants and before the year that strain *i-1* was isolated. Adjustment was then performed by standardizing the metrics with the number of post-birth strains.
