# Supplementary material for: Life course exposures continually shape antibody profiles and risk of seroconversion to influenza
Source: PLoS Pathog. 2020 Jul 23;16(7):e1008635. doi: 10.1371/journal.ppat.1008635 (PMC7377380; doi:10.1371/journal.ppat.1008635)
Supplement: S6 Table — (DOCX) [file ppat.1008635.s024.docx]

S6 Table. Univariable logistic regressions of seroconversion to four recent strains on age and pre-existing immunity.

|  | **A/Perth/2009** | **A/Victoria/2009** | **A/Texas/2012** | **A/HongKong/2014** |
| --- | --- | --- | --- | --- |
| Age at sampling | 1.00 (0.99, 1.01) | 1.00 (0.99, 1.01) | 1.00 (0.99, 1.01) | 1.00 (0.99, 1.01) |
| Titer for strain *i* | 0.56 (0.48, 0.65)* | 0.53 (0.47, 0.60)* | 0.52 (0.46, 0.59)* | 0.65 (0.57, 0.75)* |
| Titer for strain *i - 1* | 0.81 (0.73, 0.90)* | 0.66 (0.59, 0.74)* | 0.60 (0.54, 0.67)* | 0.75 (0.67, 0.83)* |
| AUC, post-birth strains | 1.02 (0.96, 1.08) | 0.96 (0.90, 1.02) | 0.93 (0.87, 0.99)* | 0.99 (0.92, 1.06) |
| ATY, post-birth strains | 1.00 (0.95, 1.04) | 0.94 (0.90, 0.99)* | 0.91 (0.88, 0.95)* | 1.00 (0.97, 1.04) |
| W_10_, post-birth strains | 1.45 (0.68, 3.08) | 0.47 (0.22, 1.01) | 0.36 (0.17, 0.75)* | 1.13 (0.54, 2.38) |
| W_40_, post-birth strains | 1.17 (0.60, 2.29) | 0.72 (0.37, 1.41) | 0.53 (0.28, 1.00)* | 0.75 (0.38, 1.48) |
| AUC, all strains | 1.00 (0.99, 1.01) | 0.99 (0.99, 1.00)* | 0.99 (0.99, 1.00)* | 1.00 (0.99, 1.00) |
| ATY, all strains | 1.02 (0.99, 1.05) | 1.00 (0.97, 1.03) | 0.97 (0.95, 1.00)* | 1.02 (0.99, 1.05) |
| W_10_, all strains | 1.11 (0.51, 2.44) | 0.35 (0.15, 0.77)* | 0.32 (0.15, 0.64)* | 0.83 (0.41, 1.65) |
| W_40_, all strains | 1.07 (0.43, 2.66) | 0.42 (0.17, 1.05) | 0.33 (0.14, 0.75)* | 0.57 (0.25, 1.30) |
