# Supplementary material for: Life course exposures continually shape antibody profiles and risk of seroconversion to influenza
Source: PLoS Pathog. 2020 Jul 23;16(7):e1008635. doi: 10.1371/journal.ppat.1008635 (PMC7377380; doi:10.1371/journal.ppat.1008635)
Supplement: S7 Table — (DOCX) [file ppat.1008635.s025.docx]

S7 Table. Univariable analysis of predictors used to assess the association between pre-existing immunity and seroconversion to four recent strains.

|  | **Age at sampling** | **Titer for strain *i*** | **Titer for strain *i – 1*** | **nAUC** | **nW_40_** |
| --- | --- | --- | --- | --- | --- |
| **A/Perth/2009** |  |  |  |  |  |
| Age at sampling | -- | -0.01  (-0.01, 0.00) | -0.02  (-0.02, -0.01)* | -0.08  (-0.09, -0.07)* | -0.01  (-0.01, -0.01)* |
| Titer for strain *i* | -0.89  (-1.81, 0.03) | -- | 0.93  (0.88, 0.99)* | 0.64  (0.51, 0.78)* | 0.05  (0.04, 0.07)* |
| Titer for strain *i - 1* | -1.83  (-2.56, -1.09)* | 0.61  (0.57, 0.65)* | -- | 0.72  (0.62, 0.83)* | 0.06  (0.05, 0.07)* |
| nAUC | -3.30  (-3.70, -2.90)* | 0.16  (0.13, 0.20)* | 0.28  (0.24, 0.32)* | -- | 0.09  (0.08, 0.09)* |
| nW_40_ | -31.80  (-36.32, -27.29)* | 1.57  (1.20, 1.95)* | 2.75  (2.31, 3.20)* | 10.13  (9.84, 10.41)* | -- |
| **A/Victoria/2009** |  |  |  |  |  |
| Age at sampling | -- | -0.01  (-0.02, -0.00)* | -0.02  (-0.02, -0.01)* | -0.08  (-0.09, -0.07)* | -0.01  (-0.01, -0.01)* |
| Titer for strain *i* | -0.93  (-1.64, -0.21)* | -- | 0.71  (0.66, 0.76)* | 0.65  (0.55, 0.75)* | 0.05  (0.04, 0.06)* |
| Titer for strain i - 1 | -1.83  (-2.56, -1.09)* | 0.76  (0.71, 0.81)* | -- | 0.72  (0.62, 0.83)* | 0.06  (0.05, 0.07)* |
| nAUC | -3.30  (-3.70, -2.90)* | 0.27  (0.23, 0.31)* | 0.28  (0.24, 0.32)* | -- | 0.09  (0.08, 0.09)* |
| nW_40_ | -31.80  (-36.32, -27.29)* | 2.60  (2.13, 3.06)* | 2.75  (2.31, 3.20)* | 10.13  (9.84, 10.41)* | -- |
| **A/Texas/2012** |  |  |  |  |  |
| Age at sampling | -- | -0.01  (-0.02, -0.00)* | -0.01  (-0.02, -0.00)* | -0.07  (-0.08, -0.06)* | -0.01  (-0.01, -0.01)* |
| Titer for strain i | -1.32  (-2.07, -0.56)* | -- | 0.92  (0.89, 0.96)* | 0.83  (0.73, 0.92)* | 0.07  (0.07, 0.08)* |
| Titer for strain i - 1 | -1.12  (-1.84, -0.40)* | 0.84  (0.81, 0.87)* | -- | 0.79  (0.70, 0.88)* | 0.07  (0.06, 0.08)* |
| nAUC | -3.30  (-3.72, -2.89)* | 0.33  (0.29, 0.36)* | 0.34  (0.30, 0.38)* | -- | 0.09  (0.09, 0.09)* |
| nW_40_ | -32.74  (-37.00, -28.47)* | 3.03  (2.64, 3.43)* | 3.15  (2.73, 3.57)* | 9.50  (9.24, 9.75)* | -- |
| **A/HongKong/2014** |  |  |  |  |  |
| Age at sampling | -- | -0.01  (-0.01, -0.00)* | -0.01  (-0.02, -0.00)* | -0.06  (-0.07, -0.05)* | -0.01  (-0.01, -0.01)* |
| Titer for strain i | -1.35  (-2.28, -0.41)* | -- | 0.96  (0.91, 1.02)* | 0.80  (0.69, 0.91)* | 0.08  (0.06, 0.09)* |
| Titer for strain i - 1 | -1.32  (-2.07, -0.56)* | 0.63  (0.59, 0.67)* | -- | 0.74  (0.66, 0.83)* | 0.07  (0.06, 0.08)* |
| nAUC | -3.37  (-3.85, -2.89)* | 0.26  (0.22, 0.29)* | 0.37  (0.32, 0.41)* | -- | 0.10  (0.09, 0.10)* |
| nW_40_ | -33.67  (-38.34, -29.00)* | 2.33  (1.97, 2.69)* | 3.28  (2.85, 3.71)* | 9.16  (8.91, 9.41)* | -- |

Note: Columns are response variables and rows are dependent variables used in the univariable linear regression. AUC and W_40_ were calculated using post-birth strains.
