# Supplementary material for: Life course exposures continually shape antibody profiles and risk of seroconversion to influenza
Source: PLoS Pathog. 2020 Jul 23;16(7):e1008635. doi: 10.1371/journal.ppat.1008635 (PMC7377380; doi:10.1371/journal.ppat.1008635)
Supplement: S8 Table — (DOCX) [file ppat.1008635.s026.docx]

S8 Table. Comparison of demographic characteristics of participants who self-reported to have not been vaccinated against influenza.

|  | **All (N, %)** | **Never vaccinated**  **(N, %)** | **P^a^** |
| --- | --- | --- | --- |
| **Total** | 777 | 573 |  |
| **Sex** |  |  |  |
| Male | 408 (52.5) | 283 (49.4) | 0.28 |
| Female | 369 (47.5) | 290 (50.6) |  |
| **Age group, years** |  |  |  |
| < 10 | 11 (1.4) | 1 (0.2) | <0.01 |
| 10-19 | 44 (5.7) | 13 (2.3) |  |
| 20-29 | 83 (10.7) | 52 (9.1) |  |
| 30-39 | 102 (13.1) | 71 (12.4) |  |
| 40-49 | 233 (30.0) | 178 (31.1) |  |
| 50-59 | 166 (21.4) | 145 (25.3) |  |
| $\geq$ 60 | 138 (17.8) | 113 (19.7) |  |
| **Employment status** |  |  |  |
| Full Time | 237 (30.5) | 174 (30.4) | 0.05 |
| Self Employed | 97 (12.5) | 80 (14.0) |  |
| Retired | 76 (9.8) | 60 (10.5) |  |
| Student | 52 (6.7) | 15 (2.6) |  |
| Homemaker | 90 (11.6) | 74 (12.9) |  |
| Unemployed | 118 (15.2) | 85 (14.8) |  |
| Other | 107 (13.8) | 85 (14.8) |  |

**^a^** P-value was derived from Chi-square test or Fisher exact test. We compared the distributions between individuals provided or not provided blood samples for each visit separately.
