# Supplementary material for: Life course exposures continually shape antibody profiles and risk of seroconversion to influenza
Source: PLoS Pathog. 2020 Jul 23;16(7):e1008635. doi: 10.1371/journal.ppat.1008635 (PMC7377380; doi:10.1371/journal.ppat.1008635)
Supplement: S9 Table — (DOCX) [file ppat.1008635.s027.docx]

S9 Table. Associations between and pre-existing immunity and seroconversion to four recent strains, participants who reported never had been vaccinated against influenza.

|  | **A/Perth/2009** | **A/Victoria/2009** | **A/Texas/2012** | **A/HongKong/2014** |
| --- | --- | --- | --- | --- |
| **Model 1** |  |  |  |  |
| Age at sampling | 1.00 (0.99, 1.02) | 1.00 (0.98, 1.01) | 1.00 (0.99, 1.01) | 1.00 (0.99, 1.02) |
| Titer to strain $i$^a^ | 0.38 (0.29, 0.50)* | 0.48 (0.39, 0.59)* | 0.49 (0.37, 0.65)* | 0.62 (0.48, 0.80)* |
| Titer to strain $i$-*1^a^* | 1.37 (1.12, 1.69)* | 1.01 (0.83, 1.23) | 1.02 (0.80, 1.31) | 0.99 (0.81, 1.21) |
| Deviance explained | 8.9% | 15.2% | 13.1% | 4.7% |
| **Model 2** |  |  |  |  |
| Age at sampling | 1.01 (1.00, 1.03) | 1.01 (0.99, 1.02) | 1.01 (1.00, 1.03) | 1.01 (1.00, 1.03) |
| Titer to strain $i$ | 0.37 (0.28, 0.49)* | 0.45 (0.37, 0.56)* | 0.44 (0.33, 0.59)* | 0.61 (0.46, 0.78)* |
| Titer to strain $i$-*1* | 1.24 (1.00, 1.53)* | 0.93 (0.75, 1.14) | 0.94 (0.73, 1.21) | 0.90 (0.73, 1.11) |
| AUC^b^ | 1.19 (1.07, 1.32)* | 1.19 (1.06, 1.34)* | 1.25 (1.11, 1.41)* | 1.17 (1.04, 1.31)* |
| Deviance explained | 10.2% | 16.4% | 14.8% | 5.6% |
| **Model 3** |  |  |  |  |
| Age at sampling | 1.01 (1.00, 1.02) | 1.00 (0.99, 1.02) | 1.01 (0.99, 1.02) | 1.01 (0.99, 1.02) |
| Titer to strain $i$ | 0.38 (0.29, 0.50)* | 0.47 (0.38, 0.57)* | 0.45 (0.34, 0.60)* | 0.62 (0.47, 0.80)* |
| Titer to strain $i$-*1* | 1.27 (1.04, 1.58)* | 0.93 (0.76, 1.15) | 0.97 (0.76, 1.26) | 0.95 (0.77, 1.17) |
| Width, cut off 1:40^b^ | 3.75 (1.30, 11.10)* | 4.77 (1.55, 15.25)* | 5.78 (1.92, 18.01)* | 1.98 (0.68, 5.81) |
| Deviance explained | 9.7% | 16.2% | 14.4% | 4.9% |

^a^ Strain *i* refers to the strain that was examined for seroconversion, and strain *i-1* refers to the most recent strain isolated prior to strain *i*. E.g. when using seroconversion to A/Perth/2009 as outcome, strain *i* and *i-1* will be A/Perth/2009 and A/Brisbane/2007, respectively.

^b^ Metrics were calculated using titers to strains isolated after the birth of the participants and before the year that strain *i-1* was isolated. Adjustment was then performed by standardizing the metrics with the number of post-birth strains.
