# Supplementary material for: Life course exposures continually shape antibody profiles and risk of seroconversion to influenza
Source: PLoS Pathog. 2020 Jul 23;16(7):e1008635. doi: 10.1371/journal.ppat.1008635 (PMC7377380; doi:10.1371/journal.ppat.1008635)
Supplement: S10 Table — (DOCX) [file ppat.1008635.s028.docx]

**S10 Table.** Associations between pre-existing immunity and seroconversion to four recent strains after accounting for sample collection time.

|  | **Adjusted odds ratio (95% confidence interval)** | | | |
| --- | --- | --- | --- | --- |
|  | **A/Perth/2009** | **A/Victoria/2009** | **A/Texas/2012** | **A/HongKong/2014** |
| **Model 1^a^** |  |  |  |  |
| Age at sampling | 1.00 (0.99, 1.01) | 0.99 (0.98, 1.00) | 0.99 (0.98, 1.00) | 1.00 (0.99, 1.01) |
| Titer to strain i^b^ | 0.43 (0.34, 0.54)* | 0.50 (0.42, 0.60)* | 0.49 (0.39, 0.63)* | 0.64 (0.52, 0.80)* |
| Titer to strain i-*1^b^* | 1.31 (1.10, 1.55)* | 1.05 (0.88, 1.24) | 1.04 (0.83, 1.31) | 0.95 (0.81, 1.13) |
| Deviance explained | 9.1% | 13.8% | 14.8% | 7.4% |
| **Model 2^a^** |  |  |  |  |
| Age at sampling | 1.01 (0.99, 1.02) | 1.00 (0.99, 1.01) | 1.00 (0.99, 1.01) | 1.01 (0.99, 1.02) |
| Titer to strain i^b^ | 0.42 (0.33, 0.53)* | 0.47 (0.39, 0.57)* | 0.45 (0.35, 0.58)* | 0.62 (0.50, 0.77)* |
| Titer to strain i-*1*^b^ | 1.22 (1.02, 1.46)* | 0.99 (0.83, 1.18) | 0.97 (0.77, 1.22) | 0.86 (0.72, 1.03) |
| AUC^c^ | 1.13 (1.04, 1.24)* | 1.16 (1.06, 1.28)* | 1.20 (1.08, 1.32)* | 1.18 (1.07, 1.31)* |
| Deviance explained | 9.8% | 14.8% | 15.9% | 8.2% |
| **Model 3^a^** |  |  |  |  |
| Age at sampling | 1.00 (0.99, 1.01) | 0.99 (0.98, 1.00) | 0.99 (0.98, 1.00) | 1.00 (0.99, 1.01) |
| Titer to strain *i*^b^ | 0.42 (0.33, 0.54)* | 0.49 (0.40, 0.58)* | 0.48 (0.38, 0.62)* | 0.63 (0.51, 0.79)* |
| Titer to strain *i-1*^b^ | 1.28 (1.08, 1.52)* | 1.05 (0.88, 1.24) | 1.00 (0.79, 1.25) | 0.89 (0.75, 1.06) |
| Width, cut off 1:40^c^ | 2.55 (1.12, 5.81)* | 2.20 (0.90, 5.36) | 2.43 (0.98, 6.05) | 3.60 (1.50, 8.65)* |
| Deviance explained | 9.4% | 14.1% | 15.1% | 7.8% |

^a^ We also included a spline interaction term of sample time at baseline and follow up for Models 1-3. The adjusted odds ratio for the spline term are not available, therefore we did not present here. See S1 Appendix for details.

^b^ Strain *i* refers to the strain that was examined for seroconversion, and strain *i-1* refers to the most recent strain isolated prior to strain *i*. E.g. when using seroconversion to A/Perth/2009 as outcome, strain *i* and *i-1* will be A/Perth/2009 and A/Brisbane/2007, respectively.

^c^ Metrics were calculated using titers to strains isolated after the birth of the participants and before the year that strain *i-1* was isolated. Adjustment was then performed by standardizing the metrics with the number of post-birth strains.

^*^ Statistical significant level of 0.05.
