# Supplementary material for: Life course exposures continually shape antibody profiles and risk of seroconversion to influenza
Source: PLoS Pathog. 2020 Jul 23;16(7):e1008635. doi: 10.1371/journal.ppat.1008635 (PMC7377380; doi:10.1371/journal.ppat.1008635)
Supplement: Attachment — Submitted filename: PLoS_Pathogens_Fluscape_Response_5May2020.docx [file ppat.1008635.s029.docx]

# Summary

## **Reviewer #1**

Yang et al. analyze a large dataset of 777 paired serological samples to generate antibody profiles against 21 H3N2 strains, at two timepoints. The authors develop new metrics, nAUC, nW_z and nATY to measure the overall magnitude of antibody response, breadth of antibody response, and temporal center of mass of the antibody response, based on each individual’s antibody profile. Empirically, many of the study’s findings confirm results from past studies of antibody landscapes, or seroconversion, but the development of new metrics to quantify, analyze and simply talk about these complicated, multidimensional features of serological data is a significant step forward, and the importance of this work should not be taken for granted.

Overall, this is a thoughtful and well-written study. The authors have clearly done a lot of careful analysis and manage to present their findings with impressive clarity and conciseness, given how complicated it is to work with and interpret longitudinal serological data. I have a few comments, but overall, I found the study quite impressive.

## **Reviewer #2**

The manuscript describes innovative methods to quantify how antibody titres and titre rises across multiple temporally spaced influenza A(H3N2) virus strains differ between groups, in this case looking at age groups. The study was well designed to collect samples a from relatively large numbers of participants aged 2-86 Y at two time-points ~ 4-years apart, and measured serum HI antibody titres against 21 A(H3N2) viruses representing ~ 46 years of virus evolution in humans (1968 – 2014). Antibody profiles were constructed by plotting each individuals HI titres against the 21 strains ordered chronologically. The main aim of the study is to derive metrics that may be used to summarize antibody profiles at different times, and then determine how profile metrics at time 1 may impact seroconversion and titre rise. Three equations or statistics were devised to characterize antibody profiles: “height” (AUC); “width” (summed time-intervals over which titres exceeded particular thresholds); and “temporal targeting of antibodies” (average year where antibody titres are concentrated). It was clear that people aged 40-60 years had relatively restricted antibody profiles in terms of AUC and width, which were centered on strains present around the midpoint of life, but it was less clear how these metrics impacted subsequent seroconversion and titre rise. Many models and tables were presented to show that some metrics improved models, albeit modestly compared to the effect of baseline homologous titre. However, the abstract, results and discussion do not clearly convey how important metrics were in terms of effects on immune responses.

**Responses:** Thanks for your comments. Including the metric would increase the deviance explained as well as AIC of the models. We’ve revised the results (page 11 line 202-204) to emphasize this: “*We found including nAUC or nW40 improved model fit in terms of deviance explained (e.g.11% increase in deviance explained after including nAUC for A/Texas/2012; Models 1-2 in Table 1) and Akaike information criterion (AIC) (S6 Fig)*”. We also emphasize this in the abstract by adding the sentence: “*Including immunity to previously exposures would also improve the deviance explained by the models*”.

Overall this is a nice study but the manuscript needs substantial re-writing, using more concise language, to convey the results and their meaning more effectively. Particular attention should be paid to the abstract. In addition, the supporting information is not set out in any particular order. The description of the supporting information does not follow the numbering of the figures and tables. It would be easier to follow the supporting information if it was broken into sections by the issue being addressed (E.g. Effects of normalization; Factors affecting seroconversion…., with the relevant supporting text, figures and tables adjacent to each other), and if there was some text to help the reader interpret the results that are being presented in each piece of supporting information. The manuscript may also be improved by carefully re-considering which topics to focus on and include in the main manuscript rather than in the supporting information.

**Responses:** Thanks for your comments. We have substantially revised our supporting information with changing headers that can reflect the issues we addressed and removing some irrelevant results (i.e. Fig. S17, Tables S5, S6, S12, S13 in the previous version). We moved the original Figure S1 (now Figure 3) which described the association between HI titers and age at isolation or age at sampling into the main text. We also moved the mediation analyses from the supporting information to the main text (page 11-12 line 209 to 217).

## **Reviewer #3**

In this paper, authors collected serum samples of people aged 2 to 86 at two time points. The baseline sampling was done in 2009 to 2011 and follow-up was done in 2014-2015. As a result, 777 paired hemagglutination inhibition titers against 21 H3N2 strains were obtained. Antibody profiles of participants were generated and used to construct models that predict the risk of infections to recent strains. This study proposed three new metrics, AUC, Wz, ATY, aiming to determine antibody profile variation for predicting the risk of seroconversion. Using logistic regression model, effects of these metrics on seroconversion were discussed. However, the usefulness of the proposed models in predicting seroconversion is unclear.

# Major Issues

## **Reviewer #1**

**Rev1Comm1:** My primary big-picture comment is that as currently written, the manuscript does not take nearly enough advantage of the flexible formatting guidelines provided by PLOS Pathogens. The text is certainly well written, but incredibly dense, possibly to the point of being difficult to follow for a non-influenza expert. Some of the main text figure panels (e.g. 2D-I) are rich with information, but not presented in the results section at all, which is a shame, and some of the results that are presented could be clearer with a bit more elaboration. It also seems a shame that the mediation analysis (Fig. S12) isn’t mentioned until the Discussion, and buried deep in the Supplement.

**Responses:** Thanks for your comment. We’ve expanded our results section with more elaboration on the figures and some supplement results in the revision. In particular,

We have added the following sentences to describe results in Figure 2:

“*Across all 21 × 777 strain-individual combinations, the most commonly detected titer value was < 1:10 at baseline (28.0%) and 1:20 and 1:40 at follow-up (22.0% and 21.8%, respectively) (Fig 2D-E).*” (page 7 line 125-127)

“*73.9% of titers to pre-birth strains were below 1:40 at baseline (62.6% at follow-up; Fig 2A-B and G-H) and the lower titers were found to these pre-birth strains with the longer intervals from isolation to birth (S1 Fig).*” (page 8 line 131-133)

We have expanded the results with more detailed information about the mediation analysis added on page 12 line 218 to 226:

“*We did not observe a positive association between immunity to non-recent strains and seroconversion to recent strains in the univariable analysis (S6 Table), which was expected given that people with higher immunity of non-recent strains tended to have higher homologous titers to recent strains (S7 Table). We therefore performed mediation analysis under the hypothesis (S7 Fig) that pre-existing immunity to pre-exposures imposes both indirect effect mediated by titer to strain i and direct effect on seroconversion to strain i. Results suggested both AUC and nW40 had negative indirect effects and positive direct effects (except for nW40 on A/HongKong/2014) on seroconversion to strain i, which yielded non-significant or marginally negative total effects (S8 Fig).*”

**Rev1Comm2:** Can the authors elaborate a bit more on the fact that titers increased across the board, even to non-recent strains, from baseline to follow up (as shown clearly in Fig. 2)? This is presented as a result, and attributed to recent exposures. But the pattern is striking, and alternate explanations involving longer storage of the baseline samples, or storage issues affecting only the baseline samples, haven’t been discussed. The results also leave me wondering if the baseline and follow-up samples were collected at different times of year, before and after the start of influenza season, respectively, so that they can be thought of as analogous to acute and convalescent samples?

**Rev1Comm2Edit:** I’m only just now (after having read the study more than once), realizing that the baseline samples were collected shortly after the 2009 pandemic, at a time when H3N2 was not the dominant circulating subtype, and H3N2 titers had not been boosted in a while. Presumably there was a big H3N2 season prior to collection of most of the follow-up samples? This is an important point, which makes the observation that titers rose across the board make a lot more sense, and which should be made explicit in the text.

**Responses:** Thanks for raising this important issue. We collected our samples throughout the year, while the majority of baseline samples were collected before the 2010 summer season that was dominated by H3N2. We collected our follow-up samples from June 2014 to June 2015, the vast majority of which were collected after the 2014 summer season. Therefore, the majority of participants experienced at least one H3N2 dominant season and showed universal increases in their antibody profiles. We believe that the increases for the non-recent strains were due to back-boostings related to the recent exposures.

To address your comment, we indicated the sample collection time in the results (page 7 line 108 - 111):

“*Both baseline (December 4, 2009 to January 22, 2011, with 44% of serum collected after the 2010 summer season started) and follow-up (June 17, 2014 to June 2, 2015, with 94% of serum collected after the 2014 summer season started) sera were available from 777 participants aged from 2 to 86 years old (age at baseline is used throughout unless otherwise noted) [18–20].*”

We also expanded in our discussion to address this (page 13-14 line 257-266):

“*Consistent with this assumption, our findings during this four-year period suggested a nearly universal exposure to H3N2 in this cohort (Figs 2C and 4D), agreeing with observations of high H3N2 incidence during this period with particularly large numbers of cases in 2011-12 that was covered by our study period [19,20]. This is consistent with other reports that the H1N1 pandemic which occurred just before or just after the beginning of our baseline sampling lead to a period of low H3N2 incidence, followed by a large resurgence of H3N2 in the period 2011-2012 [27]. 24.1% of our participants only had two-fold increase to the tested strains, suggesting widely existing exposures but probably not infections.*”

**Rev1Comm3a:** The biological interpretation of nATY is lines 153-157 is very lucid and well-written. But I’m worried that this passage conflates the extreme case, presented for illustration (nATY tracks birth year), with a biological expectation. This extreme case could only come true if individuals developed and boosted titers only to the strain of first exposure, and were never again in their lifetimes able to generate a de novo response to new strains. We know empirically this is not how influenza immunity works, and I don’t think anyone in the field would really expect nATY to track birth year perfectly—the fact that nATY moves away from birth year a little bit each year (as people are exposed to new strains and develop some de novo responses), isn’t totally surprising and I’m not sure that this in itself logically rules out any alternatives. It would be great if the authors could clarify the biological expectations here, and elaborate a bit more on the observed patterns—does nATY asymptotically approach the unweighted average over time, or does it always remain somewhere between birth year and the unweighted average. Does nATY ever skew higher than the unweighted average?

**Responses:** Thanks for your comment and sorry for the confusion. We provided the extreme cases hoping to help people understand the concept of the nATY and what it measures. We realized it might be confusing, so we split the sentence and state our hypothesis with biological expectation in a separate sentence. We’ve also expanded the elaboration more on the patterns of nATY. Below is the revised paragraph of nATY on page 10:

“*The intent of nATY is to help us understand how immunity to strains circulating at different times contributes to the overall immune profile. At the extremes, if the antibody response was completely dominated by early infections, nATY would track with birth year. However, we hypothesized that people constantly generate updated antibody responses to the newly encountered strains during their lifetimes, and nATY would track with the midpoint of the post-birth strains (i.e. unweighted average isolation year of post-birth strains). Our empirical observations are in line with our hypothesis; baseline nATY moved away from birth year at a rate of 0.50 years for every additional year of life (0.45 years at follow-up) and tracked the midpoint of the post-birth strains (Fig 4J-K). The average nATY among people aged 40 years or older, who have been exposed to all tested strains, stayed unchanged and centered on the unweighted average isolation year of all tested strains. The follow-up nATY tended to skew 2.4 years to more recent years than the unweighted average isolation year among those people who seroconverted to the recent strains (t-test, p < 0.01).* ”

**Rev1Comm3b:** A related point, which would lay the questions above to rest: Is the black dashed line showing unweighted average isolation year missing in Fig 3 J-L? Same comment in Fig. S10. I also see this dashed line is present in Fig. S2 J-L, but I’m not sure it’s plotted correctly…here I see a flat line, but if the unweighted average only considers post-birth strains, I don’t think it should be the same for all ages.

**Responses:** Sorry for the confusion. We have provided the updated Fig 3J-L and Fig. S10 (Fig S11 in the latest version) with an unweighted average isolation year of post-birth strains indicated. You are right that the unweighted average isolation year of post-birth strains is not flat and it overlaps with the average nATY predicted from the model. The dashed line in Fig. S2 J-L is the unweighted average isolation year of all strains, so it is the same for every age. We have corrected the figure legend.

## **Reviewer #2**

**Rev2Comm1:** Timing of sample collection. Given that this study investigates factors that affect antibody titre change, the timing of sample collection, and how this may differ between individuals, could be very important, and should be taken into account. The periods over which samples were collected at baseline (2009-2011) and follow-up (2014-2015) are both long, such that potential virus exposure may differ between individuals and introduce experimental differences between antibody titre and titre-rise profiles.

**Responses:** Thanks for raising this important issue. We agree that the timing for sample collections are important to understand the changes in antibody profiles between baseline and follow-up. We performed an additional sensitivity analysis to address this issue. In the sensitivity analysis, we included an additional spline term to account for the interaction between sample time at baseline and follow-up on top of the three models that were shown in Table 1. Results from the sensitivity analysis (Table S10) suggest that our main findings are not affected by the sample time. Meanwhile, we believe the observed increases in antibody profiles for the majority of our participants could be explained by our sample time, i.e. the majority of baseline samples were collected before the 2010 summer season that was dominated by H3N2 while the vast majority of which were collected after the 2014 summer season.

To address this issue, we indicated the sample collection time in the results (page 7 line 108 - 111):

“*Both baseline (December 4, 2009 to January 22, 2011, with 44% of serum collected after the 2010 summer season started) and follow-up (June 17, 2014 to June 2, 2015, with 94% of serum collected after the 2014 summer season started) sera were available from 777 participants aged from 2 to 86 years old (age at baseline is used throughout unless otherwise noted) [18–20].*”

We also added the sensitivity analysis as Table S10 and mentioned the results in the discussion (page 14 line 272-273):

“*These findings were not affected by age, self-reported vaccination or collection time (S10-11 Figs and S5, 8-10 Tables).*”

**Rev2Comm2:** Viruses

**Rev2Comm2a:** The methods should describe how were viruses were propagated – eggs or cells and cell-type?

**Responses:** Thanks for your suggestion. We’ve added the description of the virus propagation in the methods (page 18 line 359-360): “*The 50% tissue culture infectious dose (TCID50) for each virus was determined on Madin‐Darby canine kidney (MDCK) cells [32]*”.

**Rev2Comm2b:** Clade 3c.2a viruses such as HongKong/14 agglutinate turkey red blood cells very poorly and hence assays sensitivity may be poor. Was there any validation to determine whether this could account for relatively low titres against this virus in this study? In addition, some recent viruses can agglutinate via neuraminidase, which in turn can impair detection of HI antibodies. Were virus HA and NA genes sequenced and were titres tested with and without oseltamivir to determine whether titres could be affected by NA-agglutination.

**Responses:** Thanks for pointing out this important issue. Unfortunately, we did not examine the effect of NA-agglutination on HI titers to the A/HongKong/2014 strain since this is beyond the scope of our study. The main objectives of the study are to 1) develop summary metrics that could integrate the information of antibody profiles that are often highly correlated; and 2) characterize how these profiles vary across age and time using these metrics. In this perspective, our main results on the metrics algorithms and their age patterns are not expected to be largely affected by HI titers to a single strain, which was supported by the similar age-patterns of baseline and follow-up antibody profiles (Figure 4 B&C, F&G and J&K in the latest version). So we would not anticipate our results to be substantially different even if the low titers to A/HongKong/2014 was entirely due to assay sensitivity. In addition, as you mentioned in the next comment, the co-circulation of Clade 3c.2a and 3c.3a viruses in the 2014-2015 season [[1]](https://paperpile.com/c/Vfm26W/r2vo) and our sampling time (67% of the follow-up samples were collected in 2014) could also be possible reasons for the relatively low titers to A/HongKong/2014. Again, we appreciate this important issue that you raised and we have added the following in the discussion (page 12 line 237-240) to address your concern:

“*Of note, the HI titers and rises in titers to A/HongKong/2014 were relatively lower compared to other recent strains, which may due to low assay sensitivity to detect HI titers to Clade 3c.2a strains and/or the co-circulation with the Clade 3c.3a strains (e.g. A/Switzerland/2013).”*

**Rev2Comm2c:** Clade 3c.3a viruses (Eg. A/Switzerland/9715293/13) circulated widely and were associated with large epidemics in 2014, so should be included.

**Responses:** Thanks for this comment. We prioritize our tested strains with the strains that were included in the vaccine formulation and tested to create the landscape by Fonville et al. [[2]](https://paperpile.com/c/Vfm26W/5HKK) due to feasibility. So we included A/HongKong/2014 as it was selected for the vaccine formulation. In addition, we would not anticipate that including Clade 3c.3a strains would substantially affect our main findings in terms of the development of summary metrics of antibody profiles, the age pattern of these metrics and the effects of pre-existing immunity to previous exposures on seroconversion to recent strains. In particular, even with co-circulation with Clade 3c.3a viruses which probably makes seroconversion to A/HongKong/2014 harder to detect, we were still able to find positive associations between the pre-existing immunity to previous exposures and seroconversion to A/HongKong/2014 (Table 1). We would like to test our samples to the (A/Switzerland/2013) strain but simply do not have the resources to do so now.

To address your concern, we have added the following texts in the discussion on page 12 line 237-240:

“*Of note, the HI titers and rises in titers to A/HongKong/2014 were relatively lower compared to other recent strains, which may due to low assay sensitivity to detect HI titers to Clade 3c.2a strains and/or the co-circulation with the Clade 3c.3a strains (e.g. A/Switzerland/2013).*”

And on page 16 lines 308-311:

“*First, we didn’t include strain from Clade 2c.3a, which was co-circulating with A/HongKong/2014-like strain in the 2014-2015 season [30]. Our results seem to be robust as we found similar effects of immunity to previous exposures on the seroconversion to four recent strains (Table 1).*”

**Rev2Comm3a:** Models to understand antibody profile effects on seroconverion. Why was age effect considered to be linear in the main analysis when Figure 3 indicates that effects will not be linear?

**Responses:** Thanks for your comment. Figure 3 indicates the non-linear effect of age on the summary metrics of antibody profiles, while models in Table 1 were to look at the effect of age on seroconversion to recent strains after adjusting for antibody profile effects. We hypothesized the non-linear effect of age on seroconversion is largely mediated by immunity to exposures (i.e. the metrics of the antibody profiles) (as indicated by results in Table S5) and the remaining age effect could be linear due to the declining immune functions with the increasing age. Therefore, we considered the linear effect of age on seroconversion to recent strains in the main analysis. However, results from sensitivity analyses that assumed a non-linear age effect on seroconversion to recent strains suggested that our results were robust to either choice of how to represent age (i.e. as a non-linear or linear effect) (Table S5 and Fig S13 in the latest version).

In order to address your question, we added the following texts in the method (page 25 line 483-488):

“*We considered a linear effect of age on seroconversion to recent strains after adjusting for immunity to previous exposures in the main analyses as we hypothesized the latter could account for the non-linear effect of age (Table 1 and S6 Fig). We also considered a non-linear effect of age on seroconversion to recent strains in the sensitivity analyses and found qualitatively similar results with respect to the effect of other covariates in models (S5 Table and S13 Fig).*”

**Rev2Comm3b:** Why is i-1 strain titre included in all models when this had effects on seroconversion against A/Perth/2009 but not other strains. Could the situation with regard to i-1 strain reveal more complex interactions that depend on the strains involved?

**Responses:** Thanks for your comment. There is a discussion whether infection to a strain could provide temporary protection against strains that circulated in the next few seasons [[3,4]](https://paperpile.com/c/Vfm26W/w4fk+qwHI). Therefore, we included a separate term for titer to strain i-1 in our model hoping to address this issue. We agree that there could be complex interactions related to titer to strain i-1, which may need further investigations with a specifically tailored study design.

**Rev2Comm3c:** How do the authors interpret the relative impacts of AUC, width and ATY, other than that they were all positively associated with seroconversion? Are they all independently important or just related factors that indicate whether or not there is substantial existing immunity which provided a basis for boosting?

**Responses:** Thanks for your comments. Our hypothesis is that including metrics of antibody profiles that integrate previous exposure history would further improve predictions of the risk of seroconversion after adjusting for pre-existing homologous titers to the examined strain. The central idea here is including a more complete exposure history (i.e. the antibody profiles) to predict seroconversion, and AUC, width and ATY are different ways to quantify the antibody profiles. We did exploratory study in terms of this and found that any of them would be improvement in predictions (Table1, Table S4 and Figure S6) and nAUC and nW40 seems explained slightly more deviances compared to including nW10 or nATY. But because AUC, width and ATY are highly correlated so it would be more appropriate to include one each time instead including them all at once. Our results indicates including a more complete exposure history would increase the prediction of seroconversion to risk of recent strains. The exposure history was proxied by any of the metrics we developed, but indicate there is some existing immunity that provided the basis for boosting, e.g. super responder or the competence between immune responses to antigenically relatives.

**Rev2Comm3d:** There is some attempt to understand boosting versus interference (and ceiling) effects in relation to the opposite(?) effects of homologous-strain versus prior strain titres but this is not explained in much detail (line 226-229) and is hard to follow.

**Responses:** Thanks for your comments. We have expanded the results with more detailed information about the mediation analysis. The below texts have been added on page 12 line 218-226:

“*We did not observe a positive association between immunity to non-recent strains and seroconversion to recent strains in the univariable analysis (S6 Table), which was expected given that people with higher immunity of non-recent strains tended to have higher homologous titers to recent strains (S7 Table). We therefore performed mediation analysis under the hypothesis (S7 Fig) that pre-existing immunity to pre-exposures imposes both indirect effect mediated by titer to strain i and direct effect on seroconversion to strain i. Results suggested both AUC and nW40 had negative indirect effects and positive direct effects (except for nW40 on A/HongKong/2014) on seroconversion to strain i, which yielded non-significant or marginally negative total effects (S8 Fig).*”

## **Reviewer #3**

**Rev3Comm1:** Possible effect of H1N1 epidemics should be discussed. In the panels G and H in Figure 2, there is a "saddle" where we can see low titer to the H3N2 strains isolated during 1975-1980. This low titer might be related to H1N1 pandemic of the Russian flu in 1977. This point can be discussed in somewhere in the Discussion along with the concept of original antigenic sin or antigenic seniority.

**Responses:** Thanks for your comment. We believe that the observed “saddle” in Figure 2 G and H suggested low responses of the studied population to H3N2 strains that were isolated during 1975-1980. Such low responses could be due to several possible reasons, including both low H3N2 circulation and the cohort effects on the low antibody responses to these strains.

One possible explanation is that HAI antibody responses to H3N2 are generally low among people aged 40-50s at the time of circulations (who were aged 70-80s at the time when we sampled and corresponded to the “saddle”) due to accumulation of immunity during their early lives. This hypothesis is supported by a recent study which found middle-aged people were in a perpetual state of susceptibility to 3c2.A H3N2 strains (that has been circulating since 2014) due to the mounted non-neutralizing antibodies among these people [[5]](https://paperpile.com/c/Vfm26W/ep9S). These non-neutralizing antibodies, which are plausibly not protective, could bind to HAs and prevent people from generating the updated binding antibodies to HAs from the newer strains.

Another possible explanation is due to the low H3N2 activity among the studied population during 1975-1985, which could be due to the interaction with H1N1 in 1977. However, we feel the 1977 Russian flu might not completely account for the “saddle” during 1975-1980 as we detected high responses to A/Victoria/2009 (which was circulating after the 2009 pH1N1pandemic) (Figure 2B) in our studied population and low responses to H3N2 strains during 1987-1985 when there was no H1N1 pandemic.

To address this comment, we added the following texts in the discussion (page 16 line 311-314):

“*Second, between-subtype interactions have not been incorporated into the work, especially interactions with H1N1 which might confer temporary protection against H3N2 [31]. We, however, observed inconsistent responses to H3N2 strains that were isolated after the 1977 epidemic and 2009 pandemic of H1N1 (Fig 2B and H).*”

**Rev3Comm2:** For those who were born before 1968, there is no or less chance to be exposed to H3N2 viruses in their early life compared to those born after 1968. In Figure 3J and 3K, nATY is continuously declining up to the age around 50. The decrease in nATY stopped the age around 50 maybe because of no exposure to H3N2 viruses. However, in page Page 9, Line 157-158, authors wrote “Our empirical observations are more in line with the latter hypothesis”, which assumes that "all strains circulating in one’s life were equally important". This description should be modified and the possible interpretation should be added.

**Responses:** Thanks for your comment. We agree with you that nATY stopped changing at the age of around 40 years because people aged 40 years or older have lived long enough to have been exposed to all tested strains. The unweighted isolation year of post-birth strains and all strains become the same for these people (Figure 4J-K and Figure S2J-K in the latest version). We have revised the whole paragraph as below hoping to make the results clearer:

“*The intent of nATY is to help us understand how immunity to strains circulating at different times contributes to the overall immune profile. At the extremes, if the antibody response was completely dominated by early infections, nATY would track with birth year. However, we hypothesized that people constantly generate updated antibody responses to the newly encountered strains during their lifetimes, and nATY would track with the midpoint of the post-birth strains (i.e. unweighted average isolation year of post-birth strains). Our empirical observations are in line with our hypothesis; baseline nATY moved away from birth year at a rate of 0.50 years for every additional year of life (0.45 years at follow-up) and tracked the midpoint of the post-birth strains (Fig. 3J-K). The average nATY among people aged 40 years or older, who have been exposed to all tested strains, stayed unchanged and centered on the unweighted average isolation year of all tested strains. The follow-up nATY tended to skew 2.4 years to more recent years than the unweighted average isolation year among those people who seroconverted to the recent strains (t-test, p < 0.01).*” (page 10 line 174-186)

**Rev3Comm3:** The authors claim that "we found that participants who had higher immunity to previously exposed strains were more likely to experience seroconversion to recent strains after adjusting for homologous titer" in Page 12, Line 221-223. However, it is not clear how Figure S11 and Table S10, S11 support this statement. The authors should clarify this point.

**Responses:** Sorry for the confusion. Results of Figure S11, S10 and S11 were used to motivate the mediation analysis, which further investigated the direct and indirect effect of immunity to previously exposed strains on seroconversion to recent strains. We have added a paragraph of mediation analysis in the main text (page 10 line 174-186).

# Minor Issues

## **Reviewer #1**

**Rev1Comm4:** Overall, the writing in the manuscript is excellent, clear and easy to read. The Authors Summary, however, is a bit choppy in comparison to the rest of the text—consider text edits to tighten this up on revision.

**Responses:** Thanks for your comment. We have edited the Authors Summary.

**Rev1Comm5:** At their core, AUC and W_z seem to present different ways to measure the overall magnitude of an individual’s antibody response. (i.e. how much of a high-responder is any given subject?) Is there any case where we should expect these quantities to diverge in the same individual? After developing and working with them, do the authors feel one is more useful than another?

**Responses:** Thanks for your comments. nAUC can measure the overall magnitude of an individual’s antibody and every strain that has detectable titer would contribute. Meanwhile, width would be largely affected by the number of strains that were isolated in consecutive years and all had titers above a certain threshold. Therefore, if a strain itself or its previous and/or next strain with titers below the threshold would not be counted for width. Therefore, we would expect AUC and width (especially for higher threshold) to diverge in the same individual who doesn’t have a strong response to HA to a strain and its antigenically relatives, which was often observed for people aged 40-50 years old (Figure 3 B and F, C and G).

We think nAUC and width measure different properties of antibody profiles, both of which will be useful depending on the focus. nAUC could account for broad but mild cross-reactions between strains, while width is more likely to emphasize some specific responses to HA.

To address your comment, we added the below text in the discussion (page 15 line 299-301):

“*In particular, although the age patterns in AUC and widths are similar, width is more likely to capture specific responses to a HA and therefore the two metrics would diverge in a person who does not have strong responses to a strain and its antigenically relatives*”.

## **Reviewer #2**

**Rev2Comm4:** The inclusion of A/HongKong/2014 as a recent strain is questionable since there may have been limited circulation of that virus prior to follow-up sampling in 2014-2015.

**Responses:** Thanks for the comment. We agree that there were Clade 3c.3a strains circulating in 2014, but study also suggested co-circulation of Clade 3c.2a strain at that time [[1]](https://paperpile.com/c/Vfm26W/r2vo). We would not anticipate that including Clade 3c.3a strains would substantially affect our main findings. Even with co-circulation with Clade 3c.3a viruses which probably makes seroconversion to A/HongKong/2014 harder to detect, we were still able to find positive associations between the pre-existing immunity to previous exposures and seroconversion to A/HongKong/2014 (Table 1). These results were consistent with the other three recent strains.

To address your concern, we have added the following texts in the discussion on page 12 lines 237-240:

“*Of note, the HI titers and rises in titers to A/HongKong/2014 were relatively lower compared to other recent strains, which may due to low assay sensitivity to detect HI titers to Clade 3c.2a strains and/or the co-circulation with the Clade 3c.3a strains (e.g. A/Switzerland/2013).*”

and on 15 lines 308-311:

“*First, we didn’t include strain from Clade 2c.3a, which was co-circulating with A/HongKong/2014-like strain in the 2014-2015 season [29]. Our results seem to be robust as we found similar effects of immunity to previous exposures on the seroconversion to four recent strains (Table 1)*”.

**Rev2Comm5:** The interpretation of width is somewhat limited due to the imposition of thresholds for defining the strains against which a response is detected. Can this metric be additionally weighted by titre for each strain? For example if the titre is 20 instead of 40 the width is increased by half that time interval instead of the full time interval.

**Responses:** Thanks for the comment. We used the width aiming to characterize the proportion of time when titers were above a threshold, and we agree that it would partially be affected by the imposition of threshold, especially when the titers of two consecutive strains are both above or below the threshold (the first two conditions of the equation below, which is also shown Page 20 Line 432 in the main text):

However, when it fits the last two conditions, a strain with higher time would contribute to a larger fraction of time. For example, when calculate for width above 40, if the titer for a strain i was 10 (i.e. y_i,j,v_ = 1) and titer for strain i+1 was 80 (i.e. y_i+1,j,v_ = 4), then it applied the third condition of the equation. W_40,j,v_(t_i_, t_i+1_) for this scenario will be 1/3 of the time interval between strain i and i+1 were isolated. When the titer for strain i+1 was 160 (i.e. y_i+1,j,v_ = 5), W_40,j,v_(t_i_, t_i+1_) will be 1/2 of the time interval between strain i and i+1 were isolated.

**Rev2Comm6:** Multiple statements in the abstract are ambiguous as follows:

**Rev2Comm6a:** Line 38 – should qualify the time period (~ 4 Y) over which 97.8% had a titre rise.

**Responses:** Revised, thanks.

**Rev2Comm6b:** Line 40 – what does “recent strains exhibited the greatest variation” mean? Greatest titer rise,variation between age groups?

**Responses:** Revised, thanks.

**Rev2Comm6c:** Line 41 – qualify “adjusting for homologous titer” = at baseline?

**Responses:** Revised, thanks.

**Rev2Comm6d:** Line 42 - qualify = seroconversion against recent strains?

**Responses:** Revised, thanks.

**Rev2Comm7:** Results - Some of the key findings presented relate to deficits in antibody profiles among people aged 40 – 60 years. In this regard, it would improve the manuscript if additional profiles for individuals from this age group are presented in Figure 1 or as supplementary information.

**Responses:** Thanks for this comment. We’ve added a supplementary figure (new Figure S1) of another 6 individuals aged 40 to 60 years old.

**Rev2Comm7a:** Figure 1 – it may help to tie the manuscript together if AUC, width, and ATY values are also indicated for each individual shown.

**Responses:** Thanks for this comment. We have revised Figure 1 and added a table below each panel to indicate the metric values for each shown individual.

**Rev2Comm7b:** Figure 2 – as above, it may help to tie results together if age, AUC, width and ATY are indicated for the individuals shown as examples (panels A, E, I).

**Responses:** Thanks for your suggestion. We have revised Figure 3 (now Figure 4) and its legend to address this. In particular, we indicate the age information as “*The same participant, who was aged 73 years old at baseline, is used for panel E and I*”. We indicated the value of nAUC, nW40 and nATY in panel A, E and I, respectively.

**Rev2Comm7c:** Line 111 – referring to protective titres is controversial. Although 1:40 titres may be protective, or be associated with a 50% reduction in the risk of infection, this is not universal. It would be better to just say that 95.6% of participants had titres of at least 1:40 to at least one strain, and let the readers decide what that may mean.

**Responses:** We agree that 1:40 titers would not 100% protect people from infection against influenza. We’ve revised the text as suggested “.*..95.6% of participants had titers of at least 1:40 to at least one strain at baseline*”.

**Rev2Comm7d:** Line 112: 116 – it would be more meaningful to present GMT for post-birth, rather than all strains to understand differences between time 1 and time 2, and again interpretation of this result will depend upon the time of sample collection relative to virus transmission in the community.

**Responses:** Thanks for your comment. We now present GMT for post-birth strains.

**Rev2Comm7e:** Line 117 – how was the age at time of isolation of the strain against which titers were highest determined from the multiple GAM fits? Can the GAM model R code be included in the supplement?

**Responses:** We fitted a separate GAM model to titers to each strain, which means we fitted in a total 21 GAM models. For each model, we used the titer to a strain *i* as outcome and age at the time strain *i* was isolated as predictor. Therefore, we could have the age at isolation when the titer to strain *i* is the highest from the model predictions. We will make our codes available in Github (<https://github.com/UF-IDD/Fluscape_Paired_Serology>) after publication, but below are the relevant R codes we used to fit the GAMs for Figure S1 (new Fig 3 in the latest version).

spl <- NULL # list to store predictions from GAMs

for(i in 1:nrow(st)){ # strain i

used <- data[data$strain==st$strain[i], ] # subset titers of strain i

del <- rowSums(is.na(used))

used <- used[del==0, ]

if(age.type == "sampling"){ # model that uses age at sampling, panel a-c

fml <- as.formula(paste(c("log_hi_baseline",

"log_hi_followup",

"fold_of_change")[panel], # panel = 1,2,3

" ~ s(age_baseline)"))

m <- gam(fml, data = used)

new.data <- data.frame(age_baseline = seq(x.min, x.max, 0.1))

}

if(age.type == "isolation"){ # model uses age at isolation, panel d-f

x.min <- st$year_of_isolation[i] - 2010

x.max <- min(max(used$age_at_isolation), x.max.plot)

fml <- as.formula(paste(c("log_hi_baseline",

"log_hi_followup",

"fold_of_change")[panel], # panel = 1,2,3

" ~ s(age_at_isolation)"))

m <- gam(fml, data = used)

new.data <- data.frame(age_at_isolation = seq(x.min, x.max, 0.1))

}

spl[[i]] <- cbind(new.data[,1],

predict(m, newdata = new.data))

}

**Rev2Comm7f:** Line 147-152 – This is very hard to follow. What is ratio to peak? Width is described in terms of percentage of strains against which titres were above threshold, whereas on line 149 the authors switch to fraction.

**Responses:** Sorry for the confusion. Width is in percentage and the peak width occurs in people around 15 years old. However, we found drops in widths among people aged 40 - 50 years old for both nW10 and nW40, while the drop for nW40 is more dramatic compared to the drop for nW10. In order to describe the differences in drops between nW40 and nW10 among people aged 40 - 50 years, we introduced ratio to peak, i.e. the ratio between the width at 50 years old and the width at 15 years old (the peak). We have rewritten this as follows hope to make it clearer:

“*There was a more distinct drop in nW40 among participants aged 50 years old [ratio of nW40 at 50 to nW40 at 15: 0.28 (95% CI, 0.25 to 0.29) and 0.46 (95% CI, 0.43 to 0.49) for baseline and follow-up, respectively], compared to the drop in nW10 [ratio of nW10 at 50 to nW10 at 15: 0.78 (95% CI, 0.75 to 0.79) and 0.94 (95% CI, 0.90 to 0.97)] (Fig. S3).*”

**Rev2Comm7g:** Figure S6 and S7 are included to justify that models to predict seroconversion are improved if titre to strain i, strain i-1 (variable improvement), AUC and W40 are all included, but what is the difference between Figure S6 and S7. It is clear that the values differ but there is no explanation as to why?

**Responses:** Sorry for the confusion. Figure S6 shows results for models that assume a linear effect of age. Figure S7 (Figure S13 in the latest version) shows results for models that assume a non-linear effect of age, i.e. fit a spline term of age. We’ve revised the legends for the two figures to make it clearer.

**Rev2Comm7h:** Consider substituting the term “risk” for capacity- or odds- of seroconversion to avoid the inference that factors such as a high AUC are a risk factor for being infected, when what you are really looking at is the capacity to produce antibodies.

**Responses:** Thanks for your suggestion. We have changed it to “odds” to avoid any causal inference that might rise from the term “risk”.

**Rev2Comm8:** Discussion

**Rev2Comm8a:** Line 210 – the authors suggest that antibody nautch and nWidth are relatively low among people in their 40-50s because other immune responses, which are not being measured, are preventing infections from occurring. However, their data indicates that 97.8% of people are having a titre rise which they surmise to represent infection. Please justify this inference considering the data presented, as well as data from other studies on A(H3N2) virus infection rates in different age groups.

**Responses:** Thanks for your comment. Our data indicates that 97.8% of people are having a titre rise (two-fold or more) against one or more strains and 73.7% of people were seroconverted (four-fold or more rise) to one or more strains (line 182-184). There were 24.1% of people who only had two-fold rise to our tested strains, suggesting widely existing exposures but probably not infections. We believe people who have non-HA antibodies (e.g. antibody to NA [[6]](https://paperpile.com/c/Vfm26W/Xzs2)) could also prevent people from infection and the resulting generation of specific antibodies to HAs of the newest strain they exposed during the season. As a result of this, we probably would only detect broad but mild cross-reactions to these exposed but not infected strains. The low nWidth among people aged 40-50s is likely to be the collective reflection of this phenomenon.

To make the increases in titers changes more clear, we added a the below texts in the discussion:

“*24.1% of our participants only had two-fold increase to the tested strains, suggesting widely existing exposures but probably not infections.*” (page 14 line 264-266)

**Rev2Comm8b:** Line 233 – positive effects of titres to old strains on seroconversion against recent strains were only detectable in multivariate analysis.

**Responses:** Thanks for the comment. We were trying to say that replacing the antibody profile summary metrics used in models in Table 1 to titers to an individual old strain would give the similar positive association, which is the results of multivariate analysis in Figure S4. We have revised the text to make it clearer: “*This is supported by our data that positive association can still be detected when using titer to an older strain, instead of the summary metric of antibody profiles, as the proxy of immunity to non-recent strains in models shown in Table 1 (Fig. S14).*”

**Rev2Comm9:** Supporting Information

**Rev2Comm9a:** There are many tables (S3, S4, S5, S6, S7) with different versions of models, mentioned in different parts of the manuscript. This makes it quite hard to follow what is going on, and what differs between models.

**Responses:** Thanks for your comment. In the revised version, we have removed Table S5 and S6 that are not directly related with our results. We also pointed to Table S3, S4 and S7 (Table S5 in the revised version) in methods and supplementary information with indication of the issues they addressed.

**Rev2Comm9b:** Fig S4 – The caption should state that Odds Ratios were for comparison with seroconversion against HK/1968. It is not sufficient to state this in the main text only.

**Responses:** Thanks for pointing out this. We’ve indicated in the legend with “*Strain A/HongKong/1968 was set as reference*”.

**Rev2Comm9c:** Fig S5 – what do the colours mean?

**Responses:** We added the explanation of the color meanings in the legend:

“*We divided the changes in titers into four categories, i.e. decrease (green), no change (grey), two-fold increase (light purple) and four-fold change (seroconversion, dark purple).*”

**Rev2Comm10:** Minor typographical or grammatical errors

**Rev2Comm10a:** Line 372 – the each

**Responses:** Corrected, thanks.

**Rev2Comm10b:** Line 377 – decrease should be decreased

**Responses:** Corrected, thanks.

**Rev2Comm10c:** Line 603 –among for

**Responses:** Corrected, thanks.

**Rev2Comm10d:** Line 616 -618 - of people aged 40-50 years (xxxx) of participants who

**Responses:** Corrected, thanks.

**Rev2Comm10e:** Line 644 – When fitted logistic regressions of seroconversions on recent strains with age at ….(rewrite this sentence).

**Responses:** We’ve rewritten this as: “*We fitted logistic regressions to seroconversion on recent strains and adjusted age at sampling, titer to strain i, titer to strain i-1 and non-normalized summary metric of antibody profiles. Positive associations were only found for ATY for all four strains and W40 for A/Perth/2009, A/Victoria/2009 and A/Texas/2012 (Tables S3-4)*”.

**Rev2Comm10f:** Line 656 – antigenically relatives

**Responses:** Corrected, thanks.

## **Reviewer #3**

**Rev3Comm4:** Page 5. Line 70-71: Add reference to other works such as Nachbagauer R, Choi A, Hirsh A, et al. Defining the antibody cross-reactome directed against the influenza virus surface glycoproteins. Nat Immunol. 2017;18(4):464–473. doi:10.1038/ni.3684

**Responses:** We’ve cited this work.

**Rev3Comm5:** Page 6. Line 95: The term seroconversion should be defined precisely. Use an independent sentence to define what is seroconversion.

**Responses:** Thanks for the suggestion. We’ve revised as “*We define seroconversion against strain as four-fold or greater rise in titers to that strain between baseline and follow-up, which is a commonly used indicator of influenza exposure [14]*” (page 6 line 103-105).

**Rev3Comm6:** Page 7. Line 107-108: Year 2009 has two strains (i.e. A/Perth/2009, A/Victoria/2009). The reason why two strains were included should be explained.

**Responses:** Thanks for the comment. We now add the explanation for including two strains in 2009 in the introduction section, which is right after the choice of tested strains (line 97-99):

“*We included two strains that were isolated in 2009 (i.e. A/Perth/2009 and A/Victoria/2009) to address the potential difference in the circulating strain and vaccine strain in that year.* ”

**Rev3Comm7:** Page 7. Line 110: (Fig 1, A and C) should be (Fig 1, A-C), because Fig 1 B also contains "pre-birth strains".

**Responses:** Corrected, thanks.

**Rev3Comm8:** Page 7. Line 113:GMTs of pre-birth strains should be shown in Table S2.

**Responses:** Thanks for the suggestion. We’ve included GMTs of pre-birth strains in Table S2.

**Rev3Comm9:** Page 7. Line 116-117: The authors wrote, “Participants had the highest titer to strains that were isolated within the first decade of their life (4.3 years; IQR, 2.0 to 6.9 years across strains) (Fig. S1)”. However, it is difficult confirm this description only form Fig. S1. Is it possible to have another representation to visualize this claim? It is also not clear from which table the median and IQR were derived.

**Responses:** Thanks for the comments and sorry for the confusion. We have rewrote the sentence and expanded with introduction of the method, hoping to make it clearer:

“*We fitted generalized additive models (GAM) to titers on age at isolation (i.e. age when the strain was isolated) and found the highest titers at a median age at isolation of 4.3 years (IQR, 2.0 to 6.9 years) across strains (Fig 3D-E; details in S1 Appendix).* ” (page 8 line 133-136)

We have also expanded the methods in the “Age patterns in strain-specific titers” section of S1 Appendix.

**Rev3Comm10:** Page 7. Line 117-118: It is not clear from which Table the statistics of GMTs of recent strains and that of non-recent strains for baseline and follow-up visits were derived. It seems to be Table S2. If so, the table should be referred in the body text.

**Responses:** We divided titers into two groups - recent and non-recent strains - and calculated GMTs for these two groups. As there are only two groups, we did not provide a table to this.

**Rev3Comm11:** Page 8. Line 136: "Non-normalized analysis included in SI Materials and Methods" should be "...in S1 Appendix".

**Responses:** Corrected, thanks.

**Rev3Comm12:** Page 9. Line 143:2.1 (95% CI, 1.9 to 2.4) and 1.7 (95% CI, 1.5 to 1.9)

The method used for the calculation of these confidence intervals of odds ratio should be clarified.

**Responses:** Thanks for your comment.

We have provided the method in the Method section (page 23 line 447-453): “*We used the ratio of the average nAUC among people aged 50 years old to that among people aged 20 years old, to characterize the relative reduction of nAUC among people at 50 to the peak value. We derived the 95% CI of the ratio through 1000 bootstraps. In each bootstrap, we resampled the data set of observed nAUC and age at sampling, and refitted the above-mentioned GAM. We then calculated the ratio from each model prediction. With the same method, we also calculated the ratio of the average nW among people aged 50 years old to that among people aged 15 years old, at which the average nW is the greatest.* ”

We also revised the mentioned sentence (page 9 line 155-159) as “*The average nAUC among participants aged 50 years old was estimated to be lower than that among participants aged 20 years old [ratio: 0.48 (95% CI, 0.42 to 0.53; 95% CI from 1000 bootstraps and see Methods for details) for baseline and 0.59 (95% CI, 0.53 to 0.67) for follow-up]. After the age of 70, nAUC started to increase.*” to point readers to the method section.

**Rev3Comm13:** Page 9. Line 162:cIn the statement "Fig. 2C and f", "F" should be in capital letter.

**Responses:** Corrected, thanks.

**Rev3Comm14:** Page 9. Line 163: The author wrote "73.7% showed a 4-fold or greater titer increase (seroconversion) to one or more (Fig. 2I)". However, it is difficult to confirm this proportion from Fig. 2I.

**Responses:** Thanks for your comment. We’ve removed the figure in the brackets.

**Rev3Comm15:** Page 11. Line 190-193: The sentence "Based on the impact of seroconversion and transient antibody dynamics..." is difficult for readers to understand. It would be better to break it down into smaller and simpler sentences to describe the findings.

**Responses:** Thanks for your comments. We have revised the sentences as “*A majority of participants experienced seroconversion to recent strains and a minority of participants showed decreases in antibody profiles due to the transient antibody dynamics. Therefore, we expected that...*”

**Rev3Comm16:** Page 11. Line 194: Fig. 2F could not demonstrate that the increased antibody titers were those against non-recent strains. It may be better to refer to Fig. 2C instead.

**Responses:** Thanks for your suggestion. We’ve changed it to Fig. 2C.

**Rev3Comm17:** Page 12. Line 211: Grammatical error of the phrase ".., could preventing people...".

**Responses:** Corrected, thanks.

**Rev3Comm18:** Page 12. Line 229: "detailed in S Appendix" should be "detailed in S1 Appendix"

**Responses:** Corrected, thanks.

**Rev3Comm19:** Page 14. Line 712: The author mentioned "Table S18". This should be "Table S8"

**Responses:** Corrected, thanks.

**Rev3Comm20:** Page 16. Line 295: There should be a space between "A/Wuhan/1995," and "A/Victoria/1998".

**Responses:** Corrected, thanks.

**Rev3Comm21:** Page 17. Line 327: The values are added from i=1 to M-1. It is not clear why you stop at M-1 instead of M. Please describe the reason. Is it related to the two strains in 2009?

**Responses:** We used average titers {y_{i,j,v} + y_{i+1,j,v}}/2 and the time difference {t_i+1} - {t_i} between the *i*th and *(i+1)*th strain to calculate AUC. This means that we have *M-1* time differences for *M* strains, so the values are added from *i*=*1* to *M-1*.

**Rev3Comm22:** Page 20. Line 369: GAM Detailed information of the model is needed, i.e. the distribution and link function used in GAM.

**Responses:** Thanks for your comment. We now provided the equations used to fit these GAMs on page 23 line 438-446.

**Rev3Comm23:** Page 24. Line 441-442:Citation of Francis T 1960 contains its title twice.

**Responses:** Corrected, thanks.

**Rev3Comm24:** Page 33. Table 1:The definitions of Model 1, 2, and 3 are not clear. Please describe which are Model 1, 2, and 3 in the Method Section. Explanations on how to interpret values are needed for general readers. For instance, what does it mean when values are larger than 1 or smaller than 1.

**Responses:** Thanks for your suggestions.

We provided the description of Model 1-3 in the Method section on page 25 line 481-483: “*We present results of three models that included different predictors in Table 1; only including log-titer to strain i and strain i-1 (Model 1), including log-titer to strain i and strain i-1 and (Model 2), and including log-titer to strain i and strain i-1 and (Model 3)*”.

We also provided explanations of the interpretation of the OR values in the Results section on page 11 line 207-209: “*A greater frequency of seroconversion to an examined strain is expected when the estimated odds ratio (OR) of the above-mentioned predictors greater than 1 (Table 1)*”.

**Rev3Comm25:** Page 38. Line 659: "per-existing" should be "pre-existing".

**Responses:** Corrected, thanks.

**Rev3Comm26:** Page 41. Line 716: Grammatical error of the phrase "Vaccination status of influenza seems not affect...".

**Responses:** Corrected, thanks.

**Rev3Comm27:** Page 45. Line 747-749: It seems that the coefficient was normalized by comparing to A/HongKong/1968 as written in Line 165 in Page 10. If so, the figure legend should be collected to indicate this.

**Responses:** Thanks for pointing out this. We’ve added the following in the figure legend: “*The A/HongKong/1968 strain was set as reference*”.

**Rev3Comm28:** Page 46. Line 753: "changes in tiers" should be "changes in titers".

**Responses:** Corrected, thanks.

**Rev3Comm29:** Page 46. Fig. S5. There is no explanation for the different colors in this figure.

**Responses:** We have added the color explanation in the figure legend: “*We divided the changes in titers into four categories, i.e. decrease (green), no change (grey), two-fold increase (light purple) and four-fold change (seroconversion, dark purple)*”.

**Rev3Comm30:** Page 47-48. Fig. S6-S7: Explain the difference between Figure S6 and S7. The Figures look different but AIC and BIC values are the same. Figure legends are also the same.

**Responses:** Sorry for the confusion. Figure S6 shows results for models that assume a linear effect of age. Figure S7 (Figure S13 in the latest version) shows results for models that assume a non-linear effect of age, i.e. fit a spline term of age. We’ve revised the legends for the two figures to make it clearer.

**Rev3Comm31:** Page 52. Fig. S11b: Figure legend is not clear. If each column represents an individual model as stated, it is unclear what each row within the column means.

**Responses:** Each row shows the estimated correlation between the pre-existing titer to the strain listed in the y-axis on the seroconversion of the strain listed in the x-axis, after adjusting for the pre-existing titers to the rest of strains listed in the y-axis (i.e. other rows). We’ve added the following in the figure legend hoping to make it clearer:

“*Each cell within a column represents the association between pre-existing titer to the strain listed in the y-axis on the seroconversion to strain in x-axis, after adjusting for the pre-existing titers to the rest of the twenty strains*”.

**Rev3Comm32:** Page 61. Table S1: It is not clear what Pa means.

**Responses:** Sorry for the confusion. “a” was supposed to point to the footnote that the p value is from the chi-squared test. We’ve added the footnote.

**Rev3Comm33:** Supplementary Information: There are so many supplementary figures and tables. The reviewer recommends providing only figures and tables essential for this manuscript.

**Responses:** Thanks for your comment. We have substantially revised our supplementary information as suggested. In particular, we have moved the previous Fig S1 (Fig 3 in the latest version) and mediation analysis (last paragraph of the Results in the latest version) to the main text. We have removed previous Fig S8, S16, S17 and Table S5-S6 that were not directly related to our findings. We have also revised the title of sections in the supplementary information to reflect the issues that each section addressed.

# References

1.	[Shi W, Ke C, Fang S, Li J, Song H, Li X, et al. Co-circulation and persistence of multiple A/H3N2 influenza variants in China. Emerg Microbes Infect. 2019;8: 1157–1167.](http://paperpile.com/b/Vfm26W/r2vo)

2.	[Fonville JM, Wilks SH, James SL, Fox A, Ventresca M, Aban M, et al. Antibody landscapes after influenza virus infection or vaccination. Science. 2014;346: 996–1000.](http://paperpile.com/b/Vfm26W/5HKK)

3.	[Worby CJ, Wallinga J, Lipsitch M, Goldstein E. Population effect of influenza vaccination under co-circulation of non-vaccine variants and the case for a bivalent A/H3N2 vaccine component. Epidemics. 2017;19: 74–82.](http://paperpile.com/b/Vfm26W/w4fk)

4.	[Cowling BJ, Ng S, Ma ESK, Cheng CKY, Wai W, Fang VJ, et al. Protective Efficacy of Seasonal Influenza Vaccination against Seasonal and Pandemic Influenza Virus Infection during 2009 in Hong Kong. Clinical Infectious Diseases. 2010. pp. 1370–1379. doi:](http://paperpile.com/b/Vfm26W/qwHI)[10.1086/657311](http://dx.doi.org/10.1086/657311)

5.	[Gouma S, Kim K, Weirick M, Gumina ME, Branche A, Topham DJ, et al. Middle-aged individuals may be in a perpetual state of H3N2 influenza virus susceptibility. medRxiv. 2020; 2020.01.09.20017038.](http://paperpile.com/b/Vfm26W/ep9S)

6.	[Ng S, Nachbagauer R, Balmaseda A, Stadlbauer D, Ojeda S, Patel M, et al. Novel correlates of protection against pandemic H1N1 influenza A virus infection. Nature Medicine. 2019. pp. 962–967. doi:](http://paperpile.com/b/Vfm26W/Xzs2)[10.1038/s41591-019-0463-x](http://dx.doi.org/10.1038/s41591-019-0463-x)
